# The nature and distribution of putative non-functional alleles suggest only two independent events at the origins of *Astyanax mexicanus* cavefish populations

**DOI:** 10.1186/s12862-024-02226-1

**Published:** 2024-04-01

**Authors:** Maxime Policarpo, Laurent Legendre, Isabelle Germon, Philippe Lafargeas, Luis Espinasa, Sylvie Rétaux, Didier Casane

**Affiliations:** 1 Université Paris-Saclay, CNRS, IRD, UMR Évolution, Génomes, Comportement Et Écologie, 91190 Gif-Sur-Yvette, France; 2 School of Science, Marist College, Poughkeepsie, NY USA; 3 Institut de Neuroscience Paris-Saclay, Université Paris-Saclay and CNRS, 91400 Saclay, France; 4 Université Paris Cité, UFR Sciences du Vivant, 75013 Paris, France; 5 Present Address: Zoological Institute, Department of Environmental Sciences, University of Basel, Basel, Switzerland

**Keywords:** Cavefish, Relaxed selection, Pseudogenes, Visual perception

## Abstract

**Background:**

Several studies suggested that cavefish populations of *Astyanax mexicanus* settled during the Late Pleistocene. This implies that the cavefish’s most conspicuous phenotypic changes, blindness and depigmentation, and more cryptic characters important for cave life, evolved rapidly.

**Results:**

Using the published genomes of 47 *Astyanax* cavefish from la Cueva de El Pachón, El Sótano de la Tinaja, La Cueva Chica and El Sótano de Molino, we searched for putative loss-of-function mutations in previously defined sets of genes, *i.e.*, vision, circadian clock and pigmentation genes. Putative non-functional alleles for four vision genes were identified. Then, we searched genome-wide for putative non-functional alleles in these four cave populations. Among 512 genes with segregating putative non-functional alleles in cavefish that are absent in surface fish, we found an enrichment in visual perception genes. Among cavefish populations, different levels of shared putative non-functional alleles were found. Using a subset of 12 genes for which putative loss-of-function mutations were found, we extend the analysis of shared pseudogenes to 11 cave populations. Using a subset of six genes for which putative loss-of-function mutations were found in the El Sótano del Toro population, where extensive hybridization with surface fish occurs, we found a correlation between the level of eye regression and the amount of putative non-functional alleles.

**Conclusions:**

We confirm that very few putative non-functional alleles are present in a large set of vision genes, in accordance with the recent origin of *Astyanax mexicanus* cavefish. Furthermore, the genome-wide analysis indicates an enrichment of putative loss-of-function alleles in genes with vision-related GO-terms, suggesting that visual perception may be the function chiefly impacted by gene losses related to the shift from a surface to a cave environment.

The geographic distribution of putative loss-of-function alleles newly suggests that cave populations from Sierra de Guatemala and Sierra de El Abra share a common origin, albeit followed by independent evolution for a long period. It also supports that populations from the Micos area have an independent origin. In El Sótano del Toro, the troglomorphic phenotype is maintained despite massive introgression of the surface genome.

**Supplementary Information:**

The online version contains supplementary material available at 10.1186/s12862-024-02226-1.

## Background

**Fig. 1:**
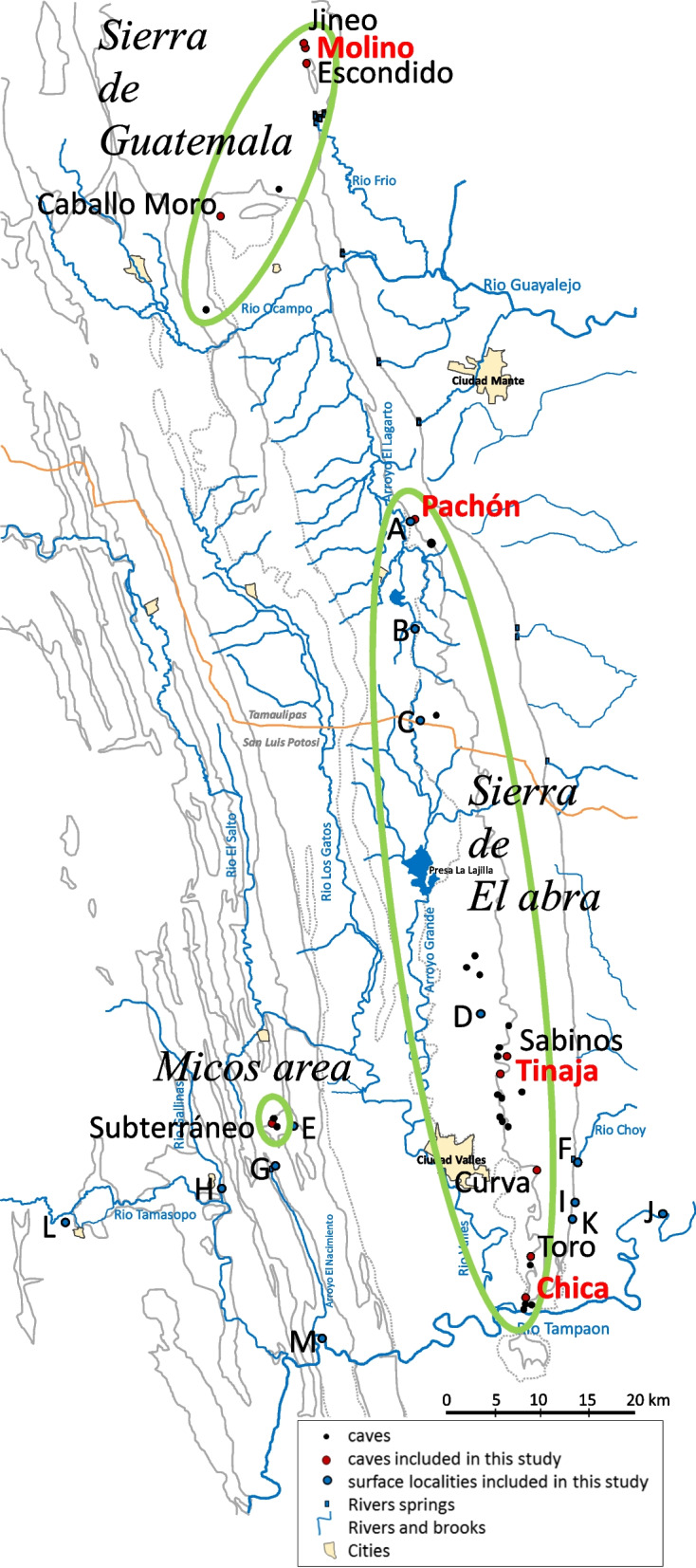
Sampling localities. Cave population names are indicated on the map. Surface fish localities: **A** Pozo de Pachón, Praxedis Guerrero; **B** Arroyo Tampemole, El Barranco; **C** Arroyo El Salvador, El Salvador; **D** Arroyo El Limoncito, Puente C85; **E** Arroyo La Pagua, Otates; **F** Nacimiento del Río Choy; **G** Nacimiento del Arroyo El Nacimiento; **H** Río Gallinas, Puente Sur Rascón; **I** Estanque Entrada Camino Taninul; **J** Río Tampaon, La Fortaleza; **K** Estanque del Cocodrilo, Taninul; **L** Arroyo Puente de Dios, Tamasopo; **M** Río Tampaon, Puente Tanchachin. In red the names of cavefish populations for which genomic data were used. Personal drawing, modified from [11]

In the species *Astyanax mexicanus*, a Mexican freshwater fish, cave and surface fish populations with strikingly different phenotypes coexist [1]. Surface fish have a wide distribution and are abundant, whereas the known cavefish are found in 33 caves [2–6] in a small area comprising three mountain ranges in Northeastern Mexico: Sierra de El Abra, Sierra de Guatemala, and Micos area (Fig. 1). The timing and the number of independent colonization events that gave rise to the cavefish populations is a matter of debate, but most recent studies point to a relatively recent origin (20,000 to 200,000 years) and few independent events (2 to 4) [7–10].

The possibility of breeding cavefish and surface fish makes *Astyanax mexicanus* an outstanding model to study genetic mechanisms underlying the evolution of some cave-associated traits such as eye loss, depigmentation, aggression loss, and sleep loss [12]. QTL analyses identified loci involved in these traits [12], but only a few studies could pinpoint changes at the DNA level [13, 14]. Different loci or mutations have been associated with similar phenotypes in some cases, demonstrating convergent evolution of some traits in different caves [12, 14, 15]. However, the fact that independent mutations are involved in the evolution of convergent traits in different cave populations does not invalidate the hypothesis of a common origin. One could imagine an ancestral population that evolved several cave-specific traits before dispersing to other caves, in which these traits continued to evolve independently in parallel with the emergence of new additional specific traits. Whereas high migration flow between distant caves is unlikely, sporadic migration over relatively long distances has been documented in other cavefishes, allowing range extension and independent evolution [16–19].

Population isolation is often studied using genetic markers assumed to be neutral, such as single-nucleotide polymorphism (SNP) and microsatellite alleles. Depending on the model assumed, if there is or is not an equilibrium between genetic drift and gene flow, it allows the estimation of demographic parameters such as effective population size and gene flow (at genetic drift / gene flow equilibrium) and isolation time (if no equilibrium is assumed). However, it is difficult to evaluate the relative effect of recent isolation and gene flow on shared polymorphism when comparing recently isolated populations. Surface and cave populations of *A. mexicanus* may share many alleles at many loci because cave populations are recent. This hypothesis is supported by an analysis of microsatellite alleles and SNPs [8] and the presence of only one pseudogene among a set of 85 vision genes in the *A. mexicanus* El Pachón cavefish reference genome; as opposed to the numerous pseudogenes found in other, more ancient cavefish species [9, 20, 21].

Here, we analyse the nature and geographic distribution of putative cave-specific loss-of-function (pLoF) mutations, alleles that most likely appeared after cave colonisation where they are neutral, and could not easily spread through surface fish to other caves because they are deleterious in surface fish. Using 47 published genomes from four different *Astyanax* cave populations, we searched for pLoF mutations in previously defined sets of genes (vision, circadian clock and pigmentation genes) and genome-wide. We identified a few pLoF in vision, circadian clock and pigmentation genes, and 533 pLoF mutations in 512 genes at the whole genome scale. Using our DNA library of wild fish from 11 different caves, we further examined the geographic distribution of 12 of these pLoF mutations. The data suggest different levels of common *versus* independent evolution among caves in the Micos area, Sierra de El Abra, and Sierra de Guatemala.

## Results

### Distribution of pLoF mutations in vision, circadian clock and pigmentation genes

In a previous study [9], we defined 63 zebrafish eye-specific genes expressed only in the eyes, together with 32 genes coding for non-visual opsins that we collectively called vision genes. We also defined two other gene sets: 42 genes involved in the circadian clock and 257 genes involved in pigmentation. In the reference genome of *Astyanax mexicanus* surface fish, we found 56, 30, 38 and 249 genes homologous to these zebrafish genes, respectively [9]. Here, using published genomic reads of 47 *Astyanax mexicanus* cavefish [7, 22], from four cave populations (9 Molino, 9 Pachón, 10 Tinaja and 19 Chica individuals), we identified pLoF mutations present in at least two individuals in these three sets of genes (Table 1). pLoF mutations correspond to loss of proper start or proper stop codons, splice site mutations, as well as premature stop codons and frameshifts identified with bcftools [23]. We only considered pLoF mutations present in protein regions conserved across teleosts, which were defined based on protein alignments with nine other annotated fish genomes. We additionally discarded premature stop codons and frameshifts located in the last 5% of the protein C-terminus region.

**Table 1:** Pseudogene frequencies in four caves among vision, circadian clock and pigmentation genes

			Caves			
Pseudogenes	Gene sets	pLoF mutations	Molino (9)	Pachón (9)	Tinaja (10)	Chica (19)
*Ψ-sagb*	Vision	Deletion		0.39	0.05	
*Ψ-cryba2a*	Vision	premature stop			0.40	0.39
*Ψ-pde6b*	Vision	deletion		0.67		
*Ψ-rh1.2*	Vision	premature stop			0.35	
*Ψ-per2*	circadian clock	insertion	1.00			
*Ψ-kif13a*	pigmentation	insertion			0.25	
*Ψ-tyrp1a*	pigmentation	deletion		0.94	0.85	0.58
*Ψ-csf1rb*	pigmentation	start loss		0.44	0.10	
*Ψ-cd63*	pigmentation	premature stop				0.26
*Ψ-drd2b*	pigmentation	deletion				0.11
*Ψ-hps4*	pigmentation	premature stop	1.00			

Sample size indicated between parentheses

First, we confirmed the pLoF mutation in the vision gene *pde6b*, found previously in the Pachón cavefish reference genome [9]. This mutation is not fixed in the Pachón population (present in 8 out 9 Pachón individuals, 4 homozygotes and 4 heterozygotes, Additional file 1). It is absent from the 3 other cave populations examined. Among vision genes, we found pLoF mutations in three additional genes (*sagb*, *cryba2a* and *rho1*), which are not present in the Pachón cavefish reference genome, and none is fixed. While the *sagb* putative non-functional allele was found in Pachón and Tinaja, the *cryba2a* putative non-functional allele was found in Tinaja and Chica, and the *rho1* putative non-functional allele was only found in Tinaja (Additional file 1). We also retrieved a recently identified pLoF mutation in the circadian clock gene *per2* [24], which is present only, and fixed, in Molino. We further detected pLoF mutations in six pigmentation genes (*csfr1b*, *kif13a*, *cd63*, *hps4*, *drd2b*, *tyrp1a*), none being fixed except *hps4*, found only in Molino (Table 1). The pLoF mutation in *tyrp1a* is present in the Pachón cavefish reference genome [9]. Thus, using our predefined gene sets, differences in the distribution of pLoF mutations emerged between Sierra de El Abra caves on the one hand (Pachón, Tinaja, and Chica) and Molino cave located in Sierra de Guatemala on the other hand.

### Genome-wide analysis of the distribution of pLoF mutations

We then extended the search for pLoF mutations genome-wide. Using genomic reads from the 47 cavefish described above, genomics reads of 15 surface fish from two surface locations (9 individuals from Nacimiento del Rio Choy and 6 from Rio Gallinas, near Rascón), as well as one *Astyanax aeneus* and one *Gymnocorymbus ternetzi* individual as outgroups, we identified a total of 533 cave specific pLoF mutations distributed in 512 genes (491 genes with 1 pLoF mutation and 21 genes with 2 pLoF mutations) (Additional file 1). Among them, there were 368 frameshifts (240 deletions and 128 insertions), 140 premature stop codons, 13 losses of the start codons, 2 losses of stop codons and 10 splice site mutations (Additional file 2: Fig. S1A). The distribution of frameshift sizes followed the expected pattern, with most indels involving one or two base pairs (Additional file 2: Fig. S1B).

**Fig. 2:**
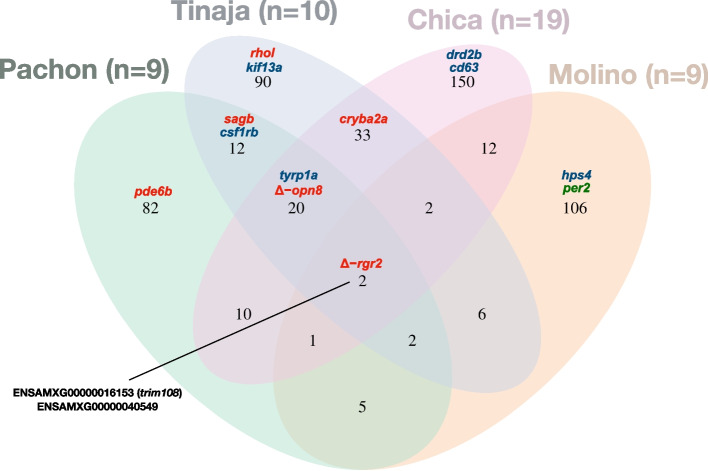
Distribution of putative non-functional alleles in four cave populations. Venn diagram using all available genomes (sample size in parenthesis). Numbers correspond to the number of pLoF identified. Genes belonging to the vision, pigmentation or circadian clock datasets and impacted by pLoF mutations are indicated (in red, blue and green respectively). Ensembl gene ID are indicated for genes that are impacted by the same pLoF mutations in the four cave populations. Cave populations where the *rgr2* and *opn8* deletions were retrieved are also indicated (*Δ-rgr2* and *Δ-opn8,* respectively)

There were 82, 90, 150 and 106 pLoF mutations that were specific to Pachón, Tinaja, Chica and Molino populations, respectively (Fig. 2). Chica had the highest count of pLoF mutations, which is not due to the higher sample size of this population, as it was still the case when sampling at random nine fish in each population (Additional file 2: Fig. S2). While 78 pLoF mutations were shared between cave pairs, only 25 were shared between 3 caves and only 2 between the 4 caves (Fig. 2). Pairwise comparisons showed that more pLoF mutations were shared between pairs of Sierra de El Abra caves (12, 10 and 33) than between a Sierra de El Abra cave and Molino cave (Sierra de Guatemala region; 5, 6 and 12) (Fig. 2). Nevertheless, 30 pLoF mutations were shared between Molino and at least one Sierra de El Abra cave (Fig. 2). The same trend was observed when considering 9 fish per population (Additional file 2: Fig S2).

### Enrichment of pLoF in genes with vision-related GO-terms

In the set of 512 pseudogenes identified above, we searched for enrichment in some GO-terms, using human and zebrafish orthologous gene names. Using orthologous zebrafish genes, we found significant enrichments in biological processes linked to eye and vision. The three most significant terms were “eye photoreceptor cell differentiation”, “peripheral nervous system neuron development” and “cellular response to UV” (Additional file 2: Table S1A). Significantly enriched terms, almost all related to eye and vision, were also found with the Phenotype GeneRIF Predicted Z-score database (Additional file 2: Table S1B).

We found that the most enriched biological process was “visual perception” with human orthologous genes using the DAVID database, but the FDR-adjusted *p*-value was not significant. Furthermore, there was a significant enrichment of genes involved in the disease “*retinitis pigmentosa*” using both OMIM and ClinVar 2019 databases (Additional file 2: Table S1C).

### Characterisation of large deletions in *opn8* and *rgr2* in cave populations

To further identify pLoF mutations due to large deletions that may have been overlooked with the above method, we used a combination of read-depth analysis and genome assembly comparison between a surface and cave fish. We identified a deletion of ~ 50 kb encompassing the 3 last exons of the non-visual opsin gene *opn8a* (gene with 4 exons) and the whole *opn8b* gene (gene with 4 exons), called *Δ*-*opn8*. These two genes are located in a genomic interval associated with differences in locomotor activity patterns between cave and surface fishes [25, 26]. The deletion was found at high frequency in Pachón (0.43), Tinaja (0.8) and Chica (0.66), but was absent in Molino (Additional file 2: Fig S3A, B).

Another deletion of ~ 3 kb genomic region containing the exons 4 and 5 of the non-visual opsin gene *rgr2* (gene with 7 exons), called *Δ*-*rgr2*, was shared at high frequency in the four populations (Molino = 1.00, Pachón = 0.94, Tinaja = 1.00 and Chica = 0.82) (Additional file 2: Fig S3C, D). Mutations in the human orthologous gene *RGR* are responsible for *retinitis pigmentosa* [27].

### Distribution of a subset of 12 pLoF mutations in 11 cave populations

We next wished to extend our analysis of the distribution of pLoF mutations to additional cavefish populations distributed in the 3 mountain ranges (Fig. 1), for which we have collected DNA samples. These included 11 caves located in Sierra de Guatemala [Jineo (n = 6 individuals), Molino (*n* = 9), Escondido (*n* = 7), Caballo moro (*n* = 2)], in Sierra de El Abra [Pachón (*n* = 55), Sabinos (*n* = 33), Tinaja (*n* = 16), Curva (*n* = 23), Toro (*n* = 30), Chica (*n* = 33)] and in Micos area [Subterráneo (*n* = 26)], together with 234 surface fish sampled in local rivers and ponds (Fig. 1). In addition to the two large deletions described above in two non-visual opsins (*opn8* and *rgr2)*, we selected 10 pLoF mutations in nine genes of particular interest: seven genes related to vision (*rh1.2, pde6b, cryba2a, impg1b, prph2b, gja8a, lim2.1*), one to pigmentation (*tyrp1a*) and one to the circadian clock (*per2*). For these 10 pLoF mutations, mutation type and position can be found in Additional file 1: LoF_list.xlsx. To note, two independent pLoF mutations were found in *per2*, one specific to Molino (called *Ψ-per2*-M, first identified with the genomic approach, see Table 1 and 2) and one specific to Tinaja (called *Ψ-per2-T*). As the latter was present in only 1 individual in the genomic data set, it was not identified initially with the genomic approach using the threshold ‘present in at-least two individuals’. Mutation frequencies estimated in Molino, Pachón, Tinaja and Chica populations from our wild-caught samples were similar to those inferred from genomic data, suggesting that both estimates were reliable (Additional file 2: Fig S4).

**Table 2:** Frequencies of 12 pseudogenes in 11 cave populations and surface fish

			*Pseudogenes*											
	L	n	*Δ*-*rgr2*	*Δ*-*opn8*	*Ψ-rh1.2*	*Ψ-cryba2a*	*Ψ-impg1b*	*Ψ-prph2b*	*Ψ-gja8a*	*Ψ-lim2.1*	*Ψ-pde6b*	*Ψ-per2-M*	*Ψ-per2-T*	*Ψ-tyrp1a*
Jineo	G	6	1.00							1.00		1.00		
Molino	G	9	1.00							1.00		1.00		
Escondido	G	7	1.00							1.00		1.00		
Caballo Moro	G	2	1.00											
Pachón	A	55	0.95	0.66	0.04		0.24	0.15			0.76			0.69
Sabinos	A	33	0.83	1.00	0.24	0.50	0.53	1.00	0.88					1.00
Tinaja	A	16	1.00	0.87	0.44	0.16	0.56	0.94	0.69				0.13	0.88
Curva	A	23	1.00	1.00	1.00		1.00	1.00	1.00					1.00
Toro	A	30	0.75	0.73		0.65	0.18	0.75	0.65					0.05
Chica	A	33	0.70	0.52		0.53								0.47
Subterráneo	M	26												
Surface		234												

*L* Location, *G* Sierra de Guatemala, *A* Sierra de El Abra, *M* Micos area, *n* sample size

The geographic distribution of pLoF mutations was very variable, from ‘specific to one cave’ to ‘present in most caves’. Moreover, when found in several caves, pLoFs primarily distributed in a geographical cluster of caves instead of being scattered in the whole cavefish distribution area (Table 2). Two pseudogenes were specific to one cave: *Ψ-pde6b* in Pachón and *Ψ-per2-T* in Tinaja. Other pseudogenes showed larger geographical areas, while none of the tested pLoF mutations was found in all 11 caves. The most widely distributed pseudogene (*Δ*-*rgr2*) was present at high frequencies in all caves except Subterráneo, in which it was absent. All other mutations were restricted to one mountain range only. The *Δ*-*opn8* and the *Ψ-tyrp1a* alleles were found in all caves in Sierra de El Abra, while *Ψ-rh1.2*, *Ψ-impg1b*, *Ψ-prph2b* and *Ψ-gja8a* were found in more restricted regions of Sierra de El Abra. On the other hand, *Ψ-per2*-M and *Ψ-lim2.1* alleles were only found in caves in the North of Sierra de Guatemala (Jineo + Molino + Escondido). Furthermore, pLoF mutations were fixed in Sierra de Guatemala, while most pLoF mutations were at high frequency (> 0.5) but not fixed in most Sierra de El Abra caves—except in Curva. None of these pLoF mutations were found in 234 surface fish or the 26 Subterráneo cavefish.

### Eye size polymorphism and surface genome introgression in the Sótano del Toro

**Fig. 3:**
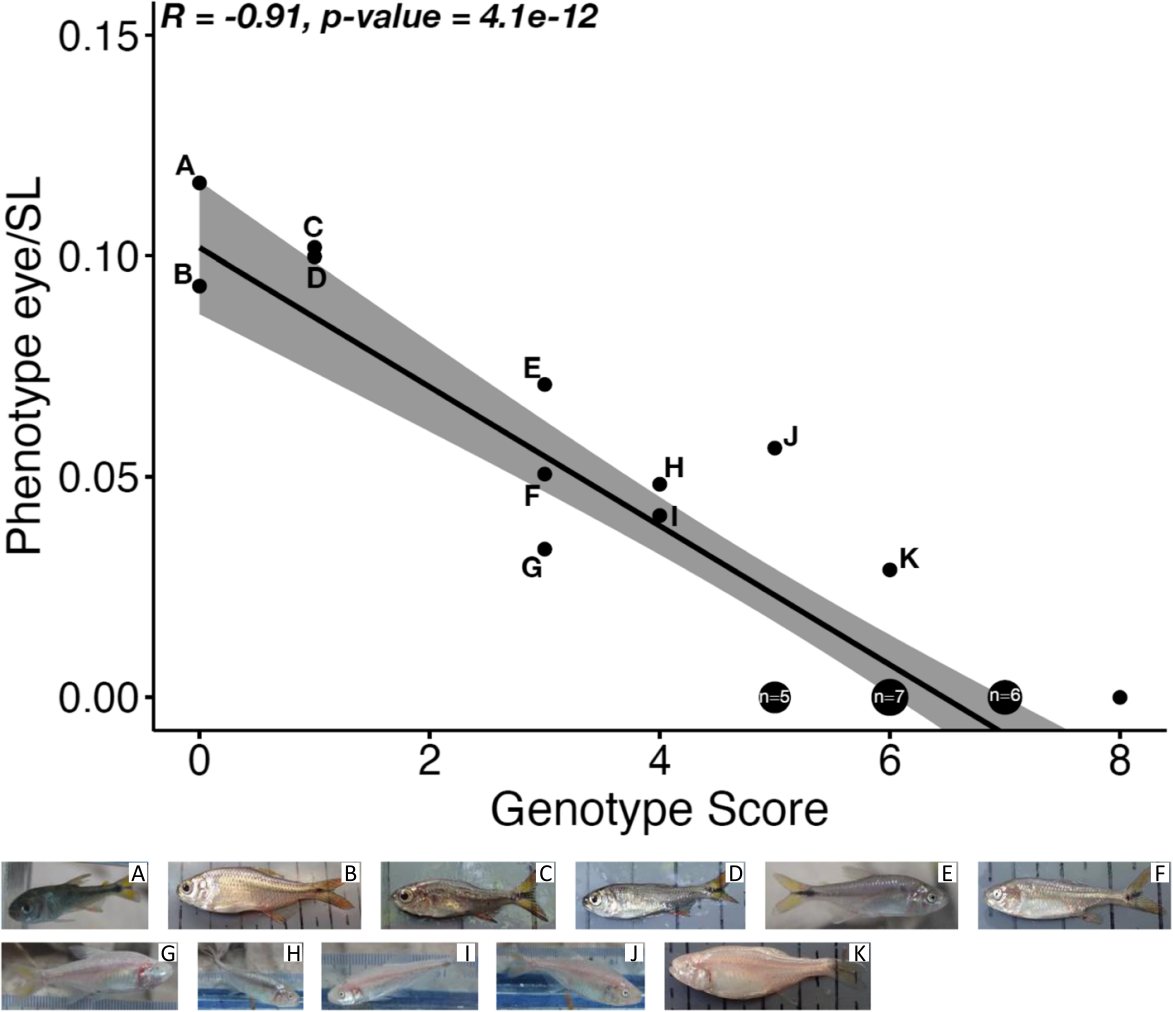
Correlation between the phenotype and genotypic score in Sótano del Toro. The phenotype score of an individual was defined as the ratio ‘eye diameter / standard length’. The genotype score was the sum of pLoF mutations. For each pLoF mutations, a score of 2 corresponds to a homozygous individual for the pLoF mutation, 1 correspond to a heterozygous individual and 0 correspond to a homozygous individual for the surface (non-mutated) allele. When different individuals had the exact same phenotype and genotype scores, we added the number of individuals in the corresponding dot. Thus, five individuals had a phenotype score of 0 and a genotype score of 5. Seven individuals had a phenotype score of 0 and a genotype score of 6, and six individuals had a phenotype score of 0 and a genotype score of 7

Finally, we sought to investigate the relationship between the level of troglomorphism, measured as the relative eye size (*i.e.*, eye diameter divided by the standard length of the fish, [28]) and the number of pLoF alleles (Additional file 1 and Additional file 2: Fig S5). This was done with samples from the Sótano del Toro (simply Toro hereafter), in which hybridization between cavefish and surface fish is frequent and gives rise to a large variability of eye sizes [5]. Although the two large deletions described above were segregating in this cave, they were not included in the analysis because our genotyping method did not allow us to differentiate between heterozygous and homozygous fish. Thus, Toro cavefish were genotyped at five loci for which segregating pLoF alleles were identified (Table 1). To have more information, we included an additional locus, the vision-associated gene *zgc:153441* (orthologous to the human gene *retinol dehydrogenase 13*), in which a 2 bp insertion was identified (Additional file 1: LoF_list.xlsx). A significant negative correlation (Pearson’s *r* = -0.91, *p*-value = 4.1e-12) was found between the relative eye size and the number of pLoF alleles (Fig. 3).

## Discussion

### Nature and age of pseudogenes segregating in caves

*Astyanax mexicanus* cavefish are a good model to study evolution in caves because they show many morphological, physiological and behavioral traits corresponding to a cave syndrome, which is often observed in animals living in this environment. The time needed to evolve many cave traits, some being adaptive and others resulting from genetic drift under relaxed selection, is a matter of debate. Several studies of the genetic polymorphism in *A. mexicanus* indicated a recent origin of cave populations (in a range of 10,000 to 200,000 years), suggesting that in this species, the cave syndrome could have evolved quickly [7, 8]. To examine further the consequences of a recent origin, we examined gene decay in several caves. First in three sets of genes involved in vision, circadian clock and pigmentation, and then at the whole genome scale. Under the hypothesis of a recent origin, we would expect that cave-specific pseudogenes would accumulate few pLoF mutations. Using 47 cavefish genomes from four different caves, our results supported this idea, as we only discovered three new vision genes with segregating putative non-functional alleles, each with only one pLoF mutation, and that were present in one or two caves. In addition, one of these pLoF mutations was previously identified in the Pachón genome assembly [9] and is only found in this population. While many vision pseudogenes can be found in other cavefish species, *A. mexicanus* data show that few vision genes are pseudogenized, thus supporting the recent evolution of blindness in this species [9, 20, 21]. Moreover, at a genome-wide scale and without a priori selection of genes involved in vision, we found an enrichment of vision-related GO-terms among 512 pseudogenes, corroborating previous studies comparing the accumulation of conservative versus radical mutations in transcripts [29]. In accordance with a recent origin of *A. mexicanus* cavefish, most pseudogenes contain only one pLoF mutation, a few of them two pLoF mutations, but none have more than two pLoF mutations [9].

### Origins of *A. mexicanus* cavefish

Another point of discussion concerns the number of independent origins of *A. mexicanus* cave populations. When they were first discovered, a different species name was given to each of them (*Astyanax jordani* Hubbs and Innes 1936 from La Cueva Chica, *A. antrobius* Alvarez 1946 from La Cueva de El Pachón, and *A. hubbsi* Alvarez 1947 form La Cueva de Los Sabinos). However, hybridization in the wild and the lab between surface and cavefish, and between cavefish from different caves suggested they belong to the same species. More recently, population genetics studies suggest gene flow and similar ages for cave populations, casting doubt on numerous independent origins of cave populations [7, 8]. Our analysis of shared pseudogenes helps to refine recent claims of a reduced number of independent origins. First, the analysis of the distribution of pseudogenes in three cave populations, at relatively large geographic distances within the Sierra de El Abra, identified many pseudogenes shared by all caves or cave pairs. This result can be best explained by a common origin resulting from a single initial colonization event, a hypothesis already proposed based on the analysis of RAPD markers [30]. Moreover, a long period of subsequent secondary independent evolution is well supported by the existence of a large number of cave-specific pseudogenes.

While there are few pseudogenes in both Sierra de El Abra and Sierra de Guatemala, it is significant that there are still several shared ones. Globally, we interpret the pattern of shared pseudogenes as evidence of a common origin of the four populations (Molino, Pachón, Tinaja, and Chica) from a single colonization event, followed with rapid isolation of Sierra de Guatemala caves from Sierra de El Abra caves. Moreover, this pattern of shared pseudogenes also suggests that the period of independent evolution of the caves is longer than the period of common evolution. In terms of biogeography, we can assume that one cavefish population evolved first, spread rapidly from one Sierra to the other, and more recently spread further within each Sierra.

To refine this scenario, we examined twelve pLoF mutations in eleven genes involved in photoreception, circadian clock and pigmentation in our library of DNA samples from 11 different *A. mexicanus* caves. The large deletion in the gene *rgr2* is shared by all cave populations in Sierra de Guatemala and Sierra de El Abra, but not with Subterráneo in the Micos area. No pLoF mutation identified in Sierra de Guatemala or Sierra de El Abra could be found in Subterráneo, strongly suggesting that the cave populations in the Micos area could have a completely distinct origin. Populations that are geo-hydrologically close to each other tend to share more markers. For example, in the Sierra de Guatemala, the three populations geographically very close in the North share two pseudogenes, which are absent in the South (Caballo Moro).

In Sierra de El Abra, while shared by several populations, most pseudogenes are not fixed, suggesting sufficient gene flow from the surface to limit their fixation in most caves. However, this is not the case in Curva, in which all pseudogenes were found fixed, which is evidence for the complete isolation of this cave [31].

The distribution of cave-specific pseudogenes suggests a separate origin of cavefish in the Micos area on the one hand and in Sierra de Guatemala + Sierra de El Abra on the other hand. In the latter region, cavefish spread rapidly from a yet unidentified cave to the whole region, leading to two well-differentiated groups: Sierra de Guatemala caves and Sierra de El Abra caves. Inside each cluster, particularly within the Sierra de El Abra, the differentiation is low. The location of the first cavity that was colonized before spreading through the underground aquifer remains unknown. Alternative explanations for shared cave specific pLoF alleles can be proposed, such as independent origin of cave populations and migrations between distant caves and/or migration of surface fish in caves bringing these mutations present at a low frequency in surface populations. When cave specific pLoF alleles are found in many caves and at high frequency, we propose that these alternatives are less likely than a common origin.

Finally, in Toro, where important gene flow from the surface is present, we observed an expected correlation between the level of phenotypic troglomorphism and the number of pLoF alleles in the genome. The continuous presence of pLoF alleles in Toro despite important surface gene flow suggests that these pLoF alleles are not easily wiped out from the cavefish genomes under underground specific environmental conditions. Regarding eye loss, the gradient of eye sizes observed in Toro individuals could be used in future studies to decipher between genes and alleles involved in controlling eye size (in individuals with small but existing eyes) and those involved in the degeneration process (in eyeless individuals).

### Remarkable convergence or common origin of cave traits?

Many studies have assumed that *Astyanax* cavefish have multiple origins with many independent colonization and convergent evolution events: 1) Micos area, 2) Sierra de Guatemala, 3) Central Sierra de El Abra (Sabinos, Tinaja, Piedras and Curva), which belong to a mitochondrial haplogroup, 4) the other caves within Sierra de El Abra which belong to another haplogroup, sometimes differentiating Pachón from Chica as yet another separate evolutionary event. Using this assumption, several studies inferred evolutionary convergences at the morphological, physiological and behavioral levels. Some were particularly remarkable.

For example, in a review of the data available on events and processes associated with eye degeneration in five caves (Sabinos, Tinaja, Curva, Pachón and Chica), no difference was found [32]. In all cavefish, when information was available, the following observations were made: smaller eye primordium, loss of ventral optic cup, lens apoptosis, continued cell division in the ciliary marginal zone, eye restoration by lens transplantation, H*sp90α* activation, *Pax6* down-regulation in optic fields, and *Hh* expansion at the embryonic midline. These similar phenotypes were assumed to result from convergence and not from a common origin. More recently, it has been proposed that the repeated evolution of eye loss in Mexican cavefish is evidence of the convergent evolution of developmental mechanisms in independently evolved Pachón and Molino populations [33]. Similarly, sleep loss in Molino, Pachón and Tinaja cavefish populations [34] and the functional and anatomical brain similarities among cavefish from Molino, Pachón and Tinaja have been interpreted as convergences [35]. Also, evolutionary convergence was proposed regarding a neural mechanism in Molino, Pachón and Tinaja cavefish lateral line system [36]. The evolution of similar regressive and constructive craniofacial traits across distinct Pachón, Tinaja and Chica cavefish populations [37] and the unique transcriptional signatures of sleep loss across Molino, Pachón and Tinaja cavefish populations were similarly interpreted. However the authors acknowledged the possibility that the traits could have evolved in their common ancestors [38]. At a genome scale, the chromatin architecture of the two cavefish morphotypes, Pachón and Tinaja, were more similar to each other than to the surface fish. These data have been interpreted as a phenotypic convergence in independently derived cavefish populations and congruent with loss of eyes and pigment, accumulation of excess fat, and insulin resistance [39].

We think that a common origin more easily explains the similarity of the cavefish syndrome in different *Astyanax* caves. Indeed, at the geographic scale of the Sierra de El Abra, this hypothesis was supported by several well-studied cave specific mutations: a *P106L* mutation in the MAO protein affecting behaviour is present in all seven Sierra de El Abra populations tested [31]; a *P211L* mutation in INSRA protein contributing to hyperglycaemia [40] is found in Pachón, Yerbaniz, Japones and Tinaja populations; an *R263Q* deleterious mutation in CRY1A is found in Pachón, Tinaja and Chica [22]. Considering the numerous putative non-functional alleles described in the present study, there is little doubt that cavefish in the whole extent of Sierra de El Abra have a common origin that explains their similar cave syndrome. A common origin does not exclude periods of independent evolution between sub-clusters of caves and within each cave, nor re-introgressions or mixing of cavefish populations after some time of independent evolution. Indeed, independent loss-of-function mutations in *oca2* are present in Molino and Pachón [14], and Pachón and Tinaja share a loss-of-function mutation in *per2* while another mutation was found in Molino [24]. Such partially independent evolution is also supported by the many cave-specific, or cave-cluster-specific, putative non-functional alleles found in the present study.

At the phenotypic level, the partial complementation restoring sight in blind cavefish larvae after crosses between different cave populations [15], the loss of schooling behaviour that is very similar between Pachón and Tinaja but shows some differences in Molino [41], or the complete loss of aggressiveness in Sierra de El Abra populations but less so in Sierra de Guatemala populations [42, 43] are also in favour of a common origin followed by independent evolution. Likewise, the observation that, among genes whose rhythmic expression is lost in at least one of three caves, 22% were arrhythmic in the three caves, was interpreted as evidence of repeated evolution of circadian clock dysregulation in independent populations [44]. It could instead suggest a common origin, explaining a large part of shared gene dysregulation, followed by independent evolution allowing both specific and “convergent” gene expression dysregulation.

## Conclusion

Altogether, the distribution of putative loss-of-function mutations in cavefish populations supports an independent origin of cavefish in the Micos area on one hand, and Sierra de Guatemala + Sierra de El Abra on the other hand. Populations in Sierra de Guatemala and Sierra de El Abra probably diverged early, while populations within Sierra de El Abra diverged later.

## Materials and methods

### Variant calling

Genomic reads from 62 *Astyanax mexicanus* fish (9 Molino, 9 Pachón, 10 Tinaja, 19 Chica, 9 Choy and 6 Rascón), one *Astyanax aeneus* individual and one *Gymnocorymbus ternetzi* individual were retrieved on NCBI (SRA accessions in Additional file 3: SRA_coverage.xlsx) and aligned to the *Astyanax mexicanus* surface fish reference genome (GCA_000372685.2) using BWA-MEM2 [45]. Duplicated reads were removed using GATK MarkDuplicates (GATK 4.2.4.1) [23]. Mean read coverage computed with SAMtools v1.15 [46] for each individual can be found in Additional file 3: SRA_coverage.xlsx.

Variant calling was performed following GATK Best Practices [47] by applying hard filters (QD < 2.0 || FS > 60.0 || MQ < 40.0 || MQRankSum < -12.5 || ReadPosRankSum < -8.0 || ExcessHet > 54.69) for SNPs and (QD < 2.0 || FS > 200.0 || ReadPosRankSum < -20.0 || ExcessHet > 54.69) for indels. Invariant sites and sites for which not all *Astyanax mexicanus* individuals were genotyped were removed. The putative impact of each variant was assessed using Ensembl Variant Effect Predictor (VEP) [48] using Ensembl v108 *Astyanax mexicanus* gene annotation.

Variants leading to putative loss-of-function (pLoF) (*i.e.*, premature stop codons, frameshifts, loss of expected start or stop codons, splice site mutations) were extracted using bcftools + split-vep [46]. Only mutations impacting the longest transcript of each gene were retained, for a total of 11,608 pLoF mutations in *Astyanax mexicanus* individuals. To reduce sequencing artifacts, we only retained 7,220 variants present in at least two individuals. We then kept 2,095 cave-specific pLoF mutations by removing variants found in surface *Astyanax mexicanus* or outgroup species (*Astyanax aeneus* and *Gymnocorymbus ternetzi*).

### Removal of artifactual pLoF variants

As inferred pLoF mutations heavily depend on the gene annotation quality, we used MAFFT v7.467 [49] to align *A. mexicanus* protein sequences (keeping the longest transcript of each gene) with orthologous sequences of 9 other teleost species retrieved using *Broccoli v1.2*: *Danio rerio* (GCF_000002035.6)*, Pygocentrus nattereri* (GCF_015220715.1)*, Electrophorus electricus* (GCF_013358815.1)*, Bagarius yarelli* (GCA_005784505.1)*, Tachysurus fulvidraco* (GCF_003724035.1)*, Ameiurus melas* (GCA_012411365.1)*, Triplophysa tibetana* (GCA_008369825.1)*, Anabarilius grahami* (GCA_003731715.1)*, Labeo rohita* (GCA_004120215.1).

Conserved regions of these alignments, *i.e.*, gene parts that are consistently annotated in the different genome assemblies [50, 51], were defined using trimAl v1.4.1 [52]. We kept 933 pLoF variants located in these conserved regions in *A. mexicanus* and removed genes for which the ensemble annotation lacked a methionine start codon.

We also removed premature stop codons and frameshifts located in the last 5% of the genes and consecutive frameshifts restoring the coding frame, for a final dataset of 533 cave-specific pLoF variants located on 512 genes.

### Distribution of pLoF mutations in vision, circadian clock and pigmentation genes

Coding sequences and genomic locations of 86 vision, 38 circadian clock and 249 pigmentation genes in the surface *Astyanax mexicanus* genome were retrieved from a previous study [9]. The vision genes dataset is defined by genes only expressed in the eye and/or the pineal complex of *Danio rerio* or *Astyanax mexicanus*, based on data retrieved on the ZFIN database (https://zfin.org/) and previous studies [29, 53]. This includes genes involved in the phototransduction cascade, as well as crystallin genes. The vision gene dataset also includes non-visual opsins, which are involved in light perception but which can be expressed in various tissues [54]﻿. The circadian clock genes dataset comprises genes involved in the circadian clock regulation [55–57]. Finally, pigmentation genes were extracted from a previous study [58]. To note, most of these genes are most likely involved in other biological processes, which is reflected by a very low proportion of these genes found with pLOF in depigmented teleost species [9].

For each of these genes, the corresponding Ensembl gene ID was retrieved on Ensembl (release 109) using a combination of blastn and the Ensembl genome browser (Additional file 1). Five genes were not retrieved on the Ensembl annotation: one vision gene (*cryaa*) and four pigmentation genes (*mc1r2*, *mgrn1a*, *shroom2a*, *ap3d1*). Furthermore, one pigmentation gene was annotated as a pseudogene on Ensembl (*mc1r1*) (Additional file 1). Vision, circadian clock and pigmentation pseudogenes were then defined using the intersection between this Ensembl gene ID list and the Ensembl gene ID of the 512 genome-wide identified pseudogenes.

### GO-term enrichment of cave pseudogenes

For each pseudogene, we retrieved *Danio rerio* and *Homo sapiens* orthologous ensembl gene name and Ensembl gene ID using the R package “biomaRt” v3.15 [59]. A total of 243 orthologous genes found in humans were used as input in the DAVID database (https://david.ncifcrf.gov/, [60]) and in EnrichR [61] to investigate diseases enrichment in the OMIM and ClinVar 2019 databases. In zebrafish, we found 335 orthologous genes, and we looked for enriched terms in the “GO Biological Process GeneRIF Predicted Z-score” and “Phenotype GeneRIF Predicted Z-score” databases implemented in FishEnrichR [61].

### Characterization of large deletions

In a previous study [9], we identified two large deletions in a Pachón cavefish genome assembly, one leading to a truncated *opn8a* gene and the absence of *opn8b* gene, the other leading to a truncated *rgr2* gene. Here, we took advantage of a new Pachón genome assembly (GCA_019721115.1) to verify and further characterize these deletions.

The genes *opn8a* (LOC111191907) and *opn8b* (LOC111197225) are located in tandem in the surface fish genome (in the genomic region NC_035899.1:44673130–44727205) and are flanked by the genes *runx2b* (LOC103028241, NC_035899.1:44525886–44575275) upstream and *mmut* (LOC103027932, NC_035899.1:44737167–44760205) downstream. We used *runx2b* and *mmut* protein sequences as tblastn [62] queries against the Pachón genome assembly to identify the corresponding region (CM033892.1:72415284–72611085). This region was extracted using samtools faidx [46], and its alignment with the surface fish region was visualized using dotter [63].

The gene *rgr2 (*LOC103043895*)* is located on the genomic region NW_019172852.1:462710–472903 in the surface genome assembly. The corresponding region in the Pachón genome assembly (CM033906.1:17152181–17158392) was extracted by using *rgr2* as a query in a tblastn against this genome. The Surface and Pachón regions were also aligned and visualized using dotter. The frequencies of these two large deletions were inferred for Molino, Tinaja, Pachón and Chica cave populations by computing the read alignment coverage in these regions for each surface and cave individual using bamCoverage [64].

### Frequencies of 10 pLoF mutations and 2 large deletions in 11 cave populations

For each cavefish or surface fish genotyped, DNA fragments containing small indels (< 7 bp) leading to frameshifts or point mutations leading to premature stop codons were amplified by PCR (primer list in Additional file 4: primer_list.xlsx). Amplicon sequencing allowed us to determine if a pLoF mutation was absent, or, if present, if the fish was heterozygous or homozygous for the variant (Additional file 2: Fig S6). For larger indels, amplification by PCR followed by a migration of amplicons on a 3% agarose gel electrophoresis allowed us to determine if an indel was absent, or, if present, if the fish was heterozygous or homozygous (Additional file 2: Fig S6).

We also genotyped fish for the two large deletions by performing two PCR for each deletion. First, a control PCR was made using primers in a non-deleted exon of *opn8a* (exon 1) or *rgr2* (exon 3) (Additional file 4: primer_list.xlsx, Additional file 2: Fig S7). A second PCR was made using a primer in putative deleted exons (exon 4 of *opn8a* or exon 5 of *rgr2*). If the second PCR led to an amplification, we inferred that the individual had at least one non-deleted allele, while if there was no amplification, the individual was considered homozygous for the deletion. This strategy did not allow us to differentiate between non-deleted homozygous and heterozygous individuals. In such case, when only one homozygous state is identifiable (here homozygosity for a deletion), we have to assume random mating to estimate the frequency of this allele in a population, which is the square root of the frequency of homozygotes.

### Phenotype and genotype scoring of Toro cavefish

A troglomorphism score (eye diameter normalized by the standard length of the fish, measured in pixels using *ImageJ* [65] was assigned to 30 wild Toro cavefish individuals photographed in the field. In this cave, cavefish cohabit with surface fish and hybrids. Eyeless specimens thus had a troglomorphism score of 0. In parallel, a genomic score was assigned to each fish: for each of the five pLoF mutations found in this population, the score was set to 2 if the fish was homozygous for the mutation, 1 if it was heterozygous, 0 if it did not carry this the mutation. To increase statistical power, fish were genotyped at a sixth locus, a vision-related gene (*zgc:15531*) for which we identified a segregating pLoF variant in the Toro population (Additional file 1, Additional file 4: primer_list.xlsx, Additional file 2: Fig S6). Scores at the six loci were summed to get a total score between 0 and 12, which was used as a proxy of the proportion of the cavefish genome (0 for surface fish and 12 for cavefish).

## Sampling authorization

*Astyanax mexicanus*: small fin clips from surface and cave morphs of *A. mexicanus* were sampled during 4 field expeditions between March 2011 and March 2017, under the auspices of the collecting permits 02241/13 (delivered to Ernesto Maldonado and S.R.), 02438/16 and 05389/17 (delivered to Patricia Ornelas-Garcia and S.R.) by the SEMARNAT (Secretaria de medio ambiente y recursos naturales) of Mexico.

### Supplementary Information

**Additional file 1: Sheet1.** Description of columns content of sheet2, sheet3, sheet4 and sheet5. **Sheet2.** List of the 533 loss-of-function variants found in cave populations. **Sheet3.** Correspondence between gene defined in Policarpo et al. 2021 and their annotation in the Ensembl database. **Sheet4.** List of 12 genotyped loss-of-function variants. The last line written in orange correspond to a loss-of-function variant that did not pass our variant filters, as only one allele was found among 12 individual from Tinaja, but that we genotyped due to its location on the *per2* gene, often found as pseudogene in cave organisms. **Sheet5.** Genotype and Phenotype score for each Toro individual.

**Additional file 2: Fig S1.** Distribution of pLoF variants. (A) Distribution of each type of pLoF variants in cave populations. (B) Frameshift sizes distribution. **Fig S2. **Distribution of pLoF alleles in four cave populations.Venn diagram using 9 genomes per population. Numbers correspond to the number of pLoF identified. Genes belonging to the vision, pigmentation or circadian clock datasets and impacted by pLoF mutations are indicated (in red, blue and green respectively). Ensembl gene ID are indicated for genes which are impacted by the same pLoF mutations in the four cave populations. Cave populations where the *rgr2* and *opn8* deletions were retrieved are also indicated (*Δ-rgr2* and *Δ-opn8* respectively). **Fig S3. **Dotterplot between surface and cave genomes. (A) *rgr2* and (B) *opn8* large deletions. X-axis correspond to the Pachón reference genome assembly (GCA_019721115.1) while Y-axis correspond to the Surface reference genome assembly (GCA_000372685.2). Genomic regions aligned are indicated along the axis. (C) and (D) represent the coverage on these regions (in the surface genome assembly) of one non-deleted individual, one heterozygous individual, and one homozygous deleted individual. **Fig S4. **Comparison of pLoF variant frequencies obtained in the WGS data and by genotyping with PCR followed by sanger sequencing or by identification in a 3.5% agarose gel. **Fig S5. **Standard length and eye diameter measures on Toro individuals. Blue lines represent the standard length and red line the eye diameter. **Fig S6.** pLoF variant genotyping. For each pLoF variant genotyped, the left panel show the corresponding genomic sequence extracted from the surface reference genome (GCA_000372685.2) as well as the primer sequences used (written in blue) and the position of the pLoF variant (highlighted in red). Primer sequences in blue can be retrieved in the Additional file 4. The right panel show examples of homozygous individuals without the pLoF variant (wt/wt), heterozygous individuals (wt/-), and homozygous individuals with the pLoF variant (-/-). **Fig S7.** Large deletion genotyping. (A) Representation of deleted regions in the cave populations. Exons of genes are represented by blue squares, and exons amplified to genotype these deletions are indicated either with blue lines (exons used as control) or red lines (exons in the deleted regions). The deletion region in cave individuals is represented with dotted lines. (B) The left panel show the expected gel pattern if an individual is not deleted. The right panel show a gel with homozygous deleted individuals (-) and with non-deleted individuals (+) for which we cannot differentiate between heterozygous or homozygous non-deleted. **Table S1.** Go-term enrichment. (A) GO-term enrichment analysis results with all human orthologous genes. Only significant GO-terms are shown. (B) GO-term enrichment analysis results with all zebrafish orthologous genes, using the “GO Biological Process GeneRIF Predicted Z-score” database. The 25 top results are shown, but neither of those are significant. (C) GO-term enrichment analysis results with all zebrafish orthologous genes, using the “Phenotype GeneRIF Predicted Z-score” database. The 25 top results are shown, but only the 21 first terms are significant.

**Additional file 3: Sheet1.** Description of columns content of sheet2 and sheet3. **Sheet2.** List of SRA accession of each individual used in this study. For Chica individuals, reads were always downloaded in two distinct files and concatenated using the bash command “cat”. **Sheet3.** Mean genome-wide coverage of genomic reads aligned against the surface reference genome (GCA_000372685.2) for each individual. These values were computed using SAMtools depth tool.

**Additional file 4: Sheet1.** Description of sheet2 columns content. **Sheet2.** List of primers used to amplify pLoF variants and the method used to genotype these variants. The list of primers used to amplify control (non-deleted) and putatively deleted exons of *opn8* and *rgr2* are also described.

**Additional file 5.** Original pictures used in Fig. 3.

## Data Availability

A bash script describing read alignment steps and variant calling, as well as a Rscript used for data analysis and related files are provided in GitHub (https://github.com/MaximePolicarpo/non-functional-alleles-Astyanax-mexicanus).
