# Supplementary material for: The nature and distribution of putative non-functional alleles suggest only two independent events at the origins of Astyanax mexicanus cavefish populations
Source: BMC Ecol Evol. 2024 Apr 1;24:41. doi: 10.1186/s12862-024-02226-1 (PMC10983663; doi:10.1186/s12862-024-02226-1)
Supplement: Supplementary file 2 — Additional file 2: Fig S1. Distribution of pLoF variants. (A) Distribution of each type of pLoF variants in cave populations. (B) Frameshift sizes distribution. Fig S2. Distribution of pLoF alleles in four cave populations.Venn diagram using 9 genomes per population. Numbers correspond to the number of pLoF identified. Genes belonging to the vision, pigmentation or circadian clock datasets and impacted by pLoF mutations are indicated (in red, blue and green respectively). Ensembl gene ID are indicated for genes which are impacted by the same pLoF mutations in the four cave populations. Cave populations where the rgr2 and opn8 deletions were retrieved are also indicated (Δ-rgr2 and Δ-opn8 respectively). Fig S3. Dotterplot between surface and cave genomes. (A) rgr2 and (B) opn8 large deletions. X-axis correspond to the Pachón reference genome assembly (GCA_019721115.1) while Y-axis correspond to the Surface reference genome assembly (GCA_000372685.2). Genomic regions aligned are indicated along the axis. (C) and (D) represent the coverage on these regions (in the surface genome assembly) of one non-deleted individual, one heterozygous individual, and one homozygous deleted individual. Fig S4. Comparison of pLoF variant frequencies obtained in the WGS data and by genotyping with PCR followed by sanger sequencing or by identification in a 3.5% agarose gel. Fig S5. Standard length and eye diameter measures on Toro individuals. Blue lines represent the standard length and red line the eye diameter. Fig S6. pLoF variant genotyping. For each pLoF variant genotyped, the left panel show the corresponding genomic sequence extracted from the surface reference genome (GCA_000372685.2) as well as the primer sequences used (written in blue) and the position of the pLoF variant (highlighted in red). Primer sequences in blue can be retrieved in the Additional file 4. The right panel show examples of homozygous individuals without the pLoF variant (wt/wt), heterozygous individuals (w [file 12862_2024_2226_MOESM2_ESM.pdf]

Additional file 2_Fig S1:

A: Distribution of each type of LoF variants in cave populations.

B: Frameshift sizes distribution.

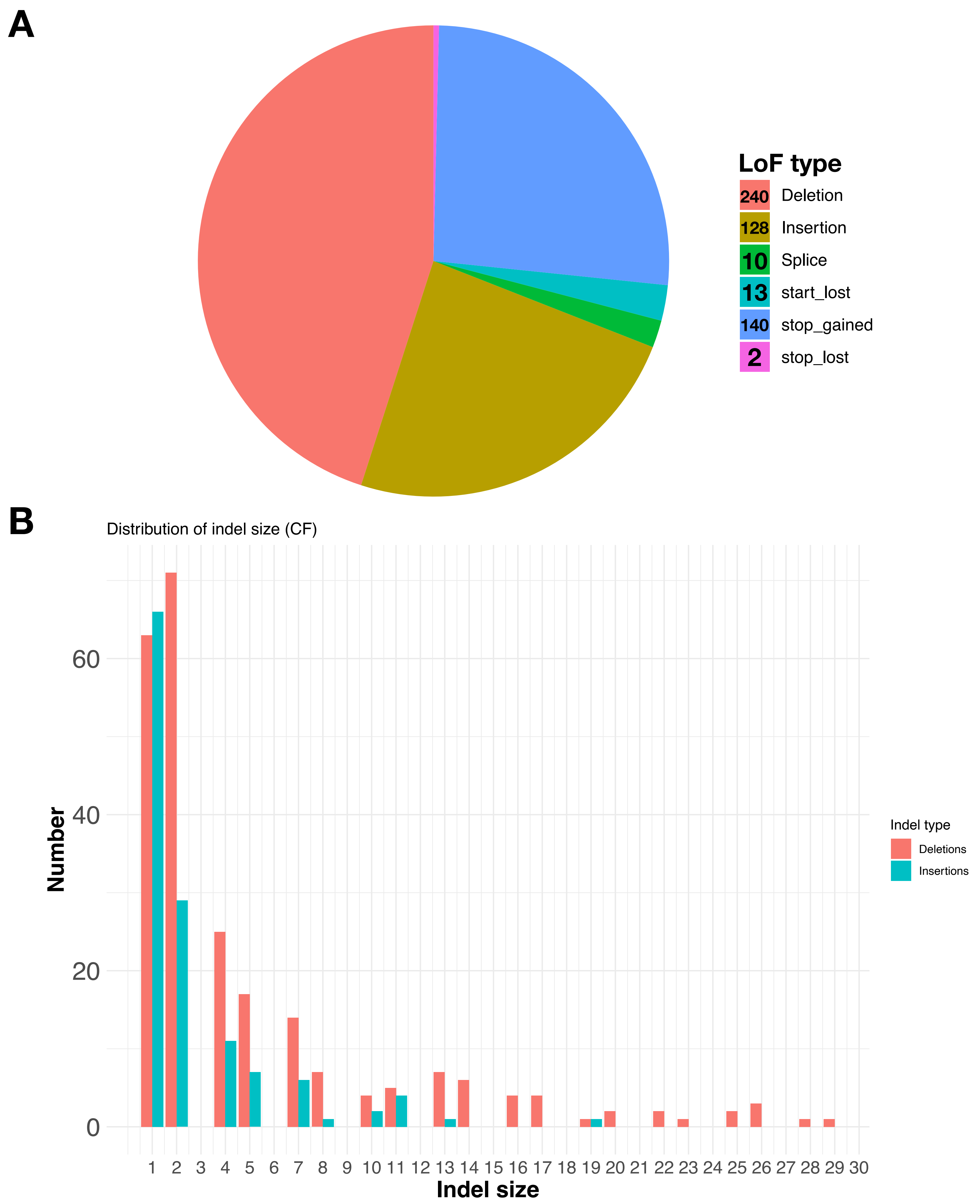

A
LoF type
240 Deletion
128 Insertion
10 Splice
13 start_lost
140 stop_gained
2 stop_lost
B
Distribution of indel size (CF)
Number
0
20
40
60
Indel size
1 2 3 4 5 6 7 8 9 10 11 12 13 14 15 16 17 18 19 20 21 22 23 24 25 26 27 28 29 30
Indel type
Deletions
Insertions

Additional file 2_Fig S2:

Distribution of putative non-functional alleles in four cave populations.

Venn diagram using 9 genomes per population. Numbers correspond to the number of pLoF identified. Genes belonging to the vision, pigmentation or circadian clock datasets and impacted by pLoF mutations are indicated (in red, blue and green respectively). Ensembl gene ID are indicated for genes which are impacted by the same pLoF mutations in the four cave populations. Cave populations where the *rgr2* and *opn8* deletions were retrieved are also indicated (*Δ-rgr2* and *Δ-opn8* respectively).

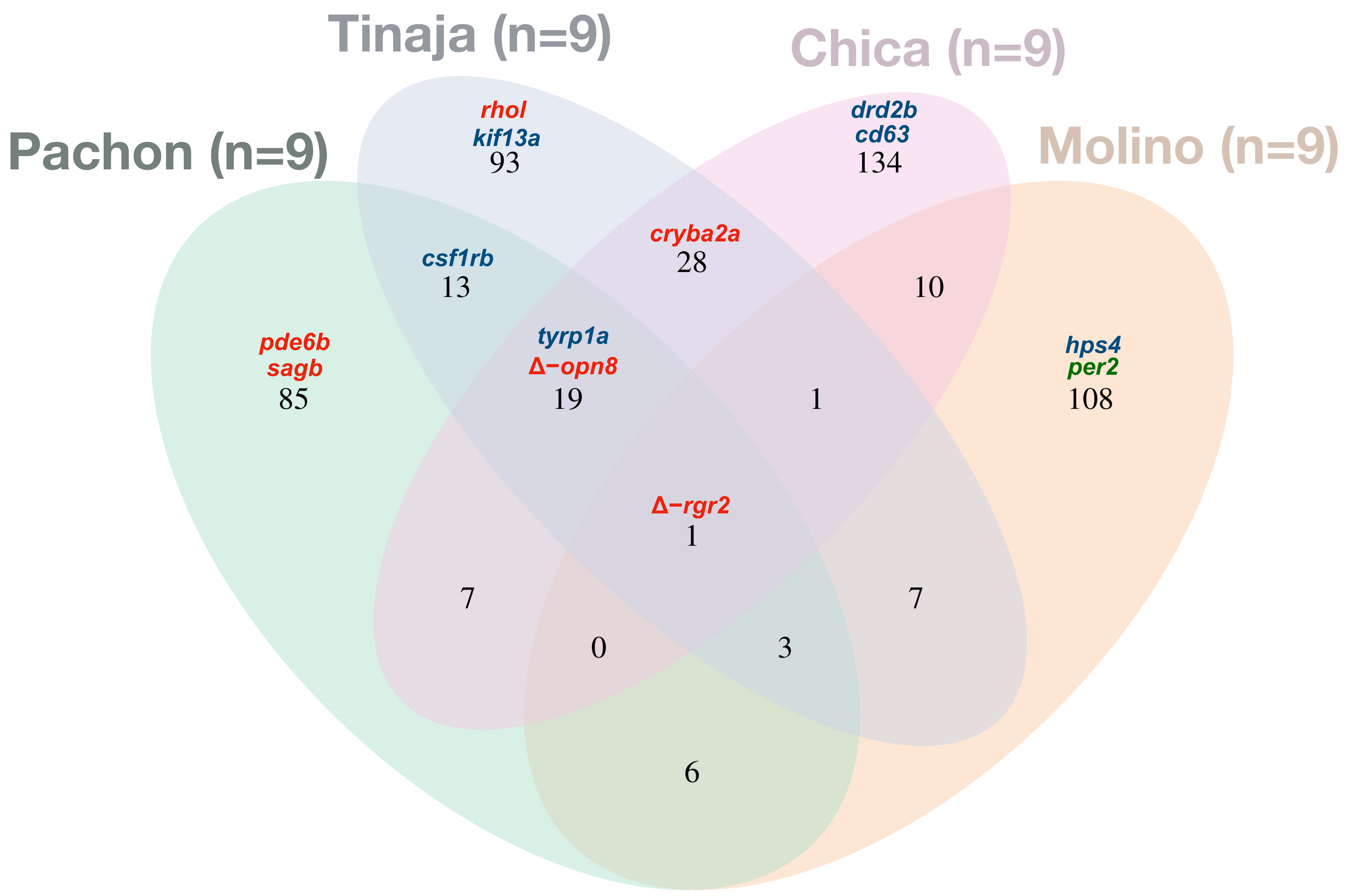
Tinaja (n=9)
Chica (n=9)
Pachon (n=9)
Molino (n=9)
rhol
kif13a
93
drd2b
cd63
134
cryba2a
28
csf1rb
13
10
pde6b
sagb
85
tyrp1a
Δ−opn8
19
1
hps4
per2
108
Δ−rgr2
1
7
7
0
3
6

Additional file 2_Fig S3:

Dotterplot between surface and cave genomes showing (A) *rgr2* and (B) *opn8* large deletions. X-axis correspond to the Pachón reference genome assembly (GCA_019721115.1) while Y-axis correspond to the Surface reference genome assembly (GCA_000372685.2). Genomic regions aligned are indicated along the axis.

(C) and (D) represent the coverage on these regions (in the surface genome assembly) of one non-deleted individual, one heterozygous individual, and one homozygous deleted individual.

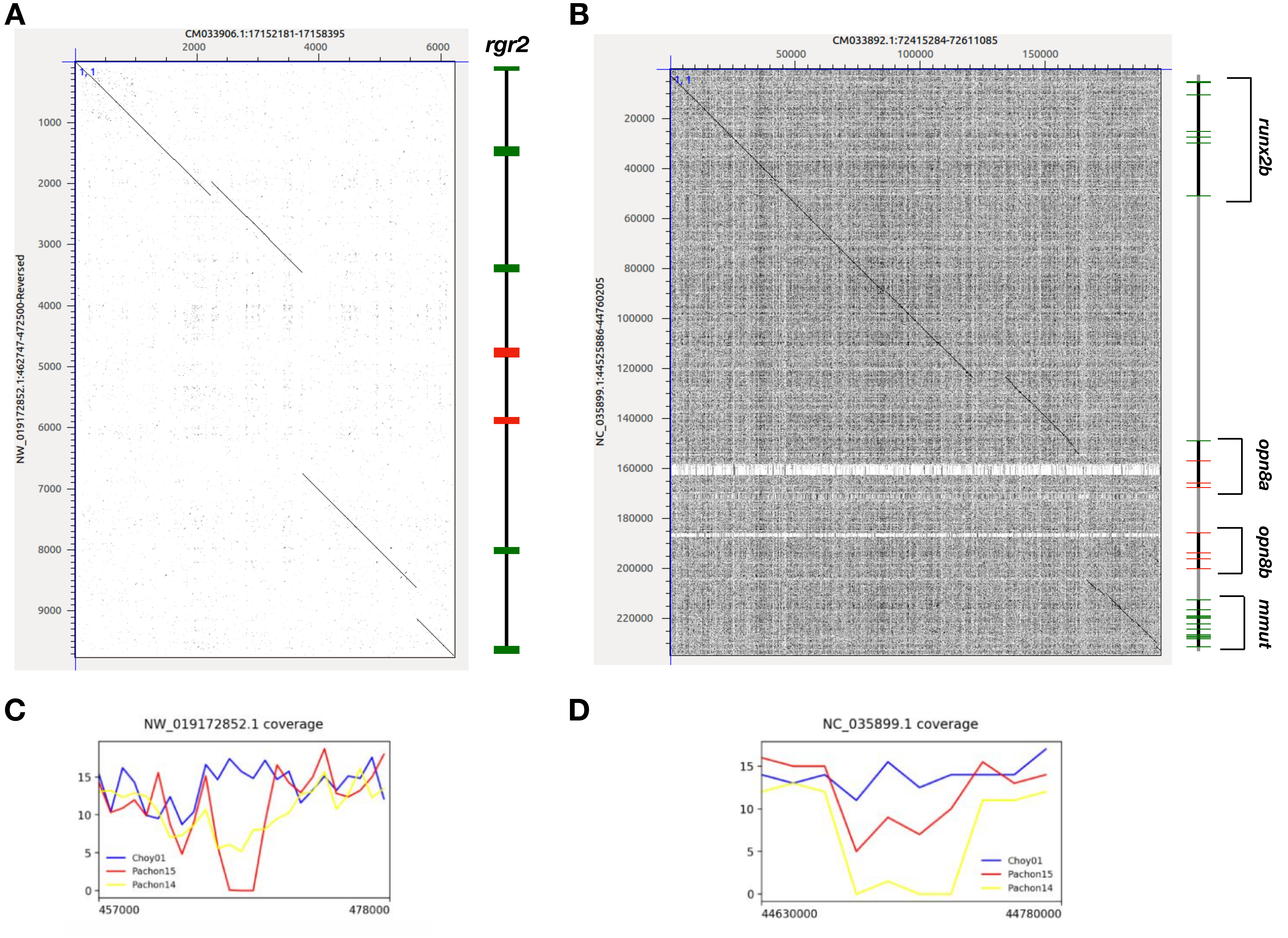
A
CM033906.1:17152181-17158395
NW_019172852.1:462747-472500-Reversed
rgr2
B
CM033892.1:72415284-72611085
NC_035899.1:44525886-44760205
runx2b
opn8a
opn8b
mmut
C
NW_019172852.1 coverage
Choy01
Pachon15
Pachon14
457000
478000
D
NC_035899.1 coverage
Choy01
Pachon15
Pachon14
44630000
44780000

Additional file 2_Fig S4:

Comparison of pLoF variant frequencies obtained in the WGS data and by genotyping with PCR followed by sanger sequencing or by identification in a 3.5% agarose gel.

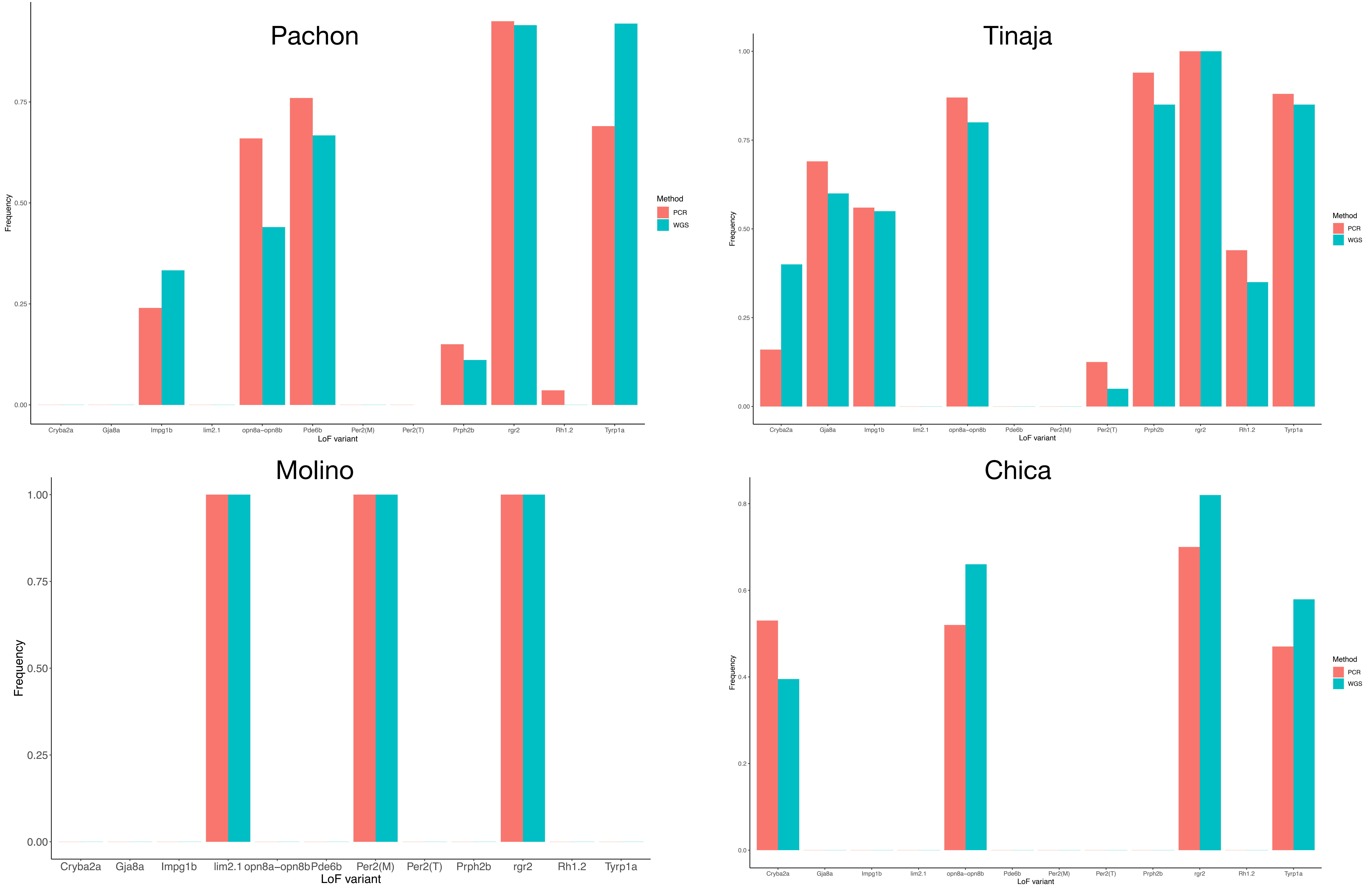

Pachon
Tinaja
Molino
Chica
Frequency
LoF variant
Method
PCR
WGS
0.00
0.25
0.50
0.75
1.00
0.0
0.2
0.4
0.6
0.8
Cryba2a
Gja8a
Impg1b
lim2.1
opn8a–opn8b
Pde6b
Per2(M)
Per2(T)
Prph2b
rgr2
Rh1.2
Tyrp1a

Additional file 2_Fig S5:

Standard length and eye diameter measures on Toro individuals. Blue lines represent the standard length and red line the eye diameter.

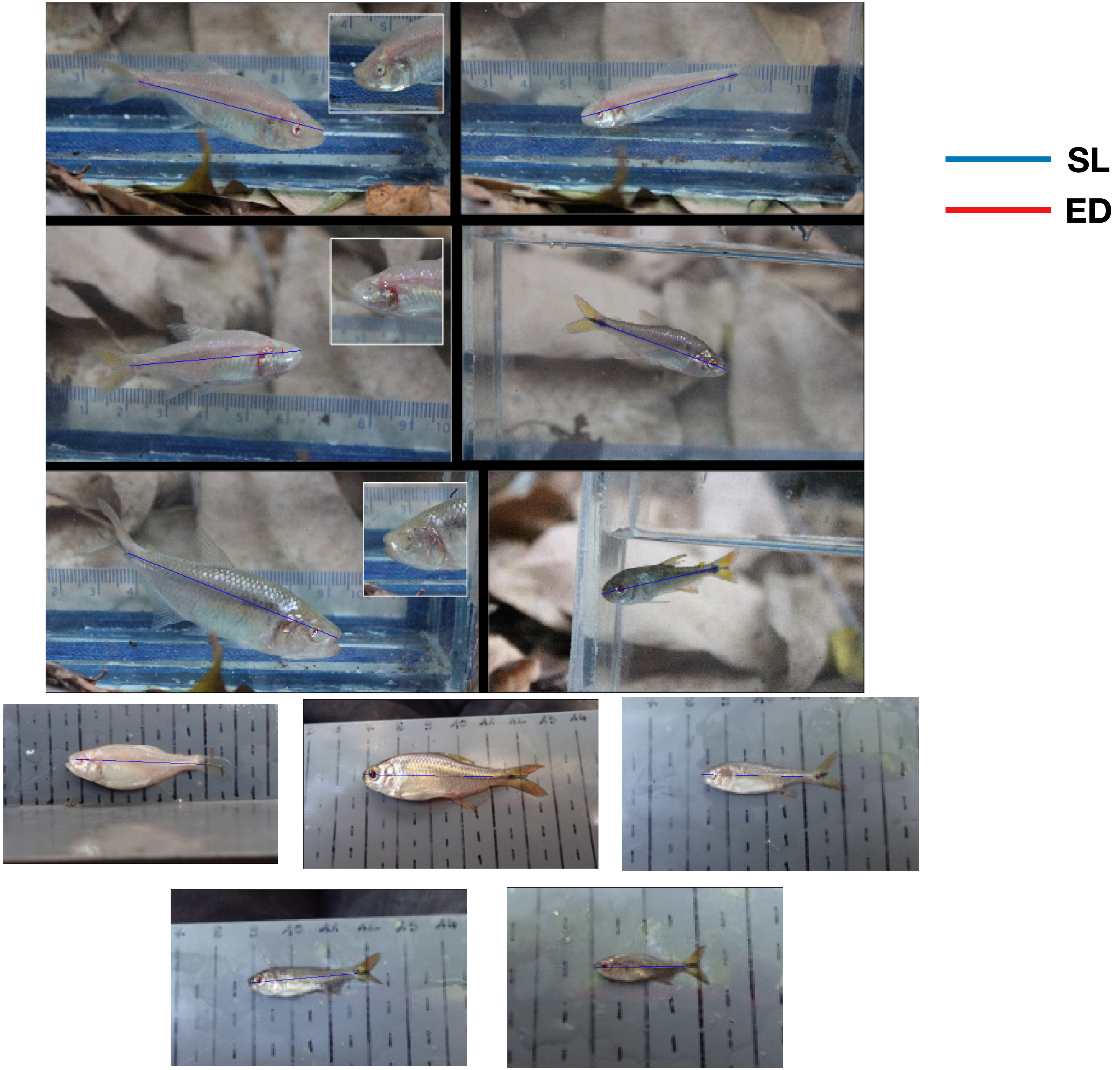
SL
ED

Additional file 2_Fig S6:

For each pLoF variant genotyped, the left panel show the corresponding genomic sequence extracted from the surface reference genome (GCA_000372685.2) as well as the primer sequences used (written in blue) and the position of the pLoF variant (highlighted in red). Primer sequences in blue can be retrieved on the additional file 4_primer_list.

The right panel show examples of homozygous individuals without the pLoF variant (wt/wt), heterozygous individuals (wt/-), and homozygous individuals with the pLoF variant (-/-).

rh1.2

Stop codon (TGG → TGA)

ATGAACGGCACG**GAGGGTCCCGACTTCTTTGTG**CCCATGTCCAATGAGACGGG
AGTGGTGAGGAGCCCCTTCGAGTACCCACAGTACTACCTGGCGGATCCATGGG
TGTTCAAAGCGATCGCGGCGTACATGTTCTTCCTTATAATCACGGGCTTCCCCA
TCAATGCCTTTACGCTCCACGTCACTATCGAGCACCAGAAGCTGAGGACTCCG
CTCAACTACATCCTGCTGAATCTGGCCACAGCCAACCTTTTCATGGTGATTGGC
GGTTTTACCACCACCATGTACACAGCCCTGCATGGCTACTTCATCTTTGGCCAT
GCTGGCTGCAACTTGGAAGGATTCTTCGCCACTCTCGGTGGTGAGATCTCGCT
TTGGTCACTGGTGGTCCTTGCTGTGGAAAGGTGGATGGTGGTCTGCAAGCCTC
TCAAAACCTTTCGCTTCCGCCAGATCCACGCCTGCCTCGGAGTCGGCTTTTCT
TGGATGATGGCCTTCTCTTGTGCGCTGCCACCTCTTCTGGAATGGTCTCGCTTC
ATCCCTGAGGGCTTACAGTGCTCTTGCGGAGTCGACTACTACACCGAAAGTCC
TCATCTCTTCAACGAGTCCTTCGTCATCTACATGTTCGCGGTTCACTTCACCAT
CCCGCTGACCGTCATCAGCTTTTGCTACACCTGGCTGCTCTGCACAGTCAAGG
CAGCGGCGGCCGCGCAGCAGGAGTCTGAGACTACGCAGAGGGCAGAGCGAG
AGGTCACCCGCATGGTCATCGTGATGATCGTGGCGTTCCTTATTTGCTGGCTGC
CATACGCAAGCATTGCCTGGTACATCTTCACACATCCCGGAATCGTCGTCAAC
CCCCTCTTCATGACCATTCCCTCTTTCTTCGCCAAGAGCTCAACGCTCTACAA
CCCTGTAATCTACGTCTGCATGAACAAGCCGTTCAGAGACTGCATGATCCGCA
CCATCTGCGGTGGCGAGAATCCGTTTGAGAAGCCGGATGAGTCCACCACCTCC
AGCTCCTCCAAGACGGAAG**CCTCCTCTGTGTCCTCCAGT**TCAGTTTCTCCTGA
ATAA

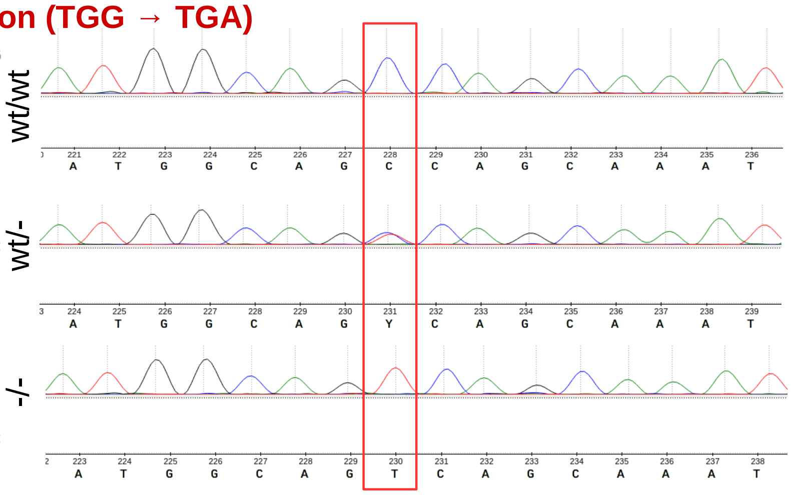

tyrp1a

1bp deletion

ACGGTTTCTTTAAAATAACAAAGTTTAATAATTGCCAAAGTTTAAAGCAA
CATATAAACACTAATGTTTTTAGTCCTCTATTGACCTCTGTGACATTTGC
AGTACTGAACCCTTATTGTATCTATTGTTATTAAAAAGTAAATACTAATAG
CATAATTCTAATAAATGATATGATTTTTTCTCCAGCCCGGCCCGTCACT
CTGACTGAGATCATCACCATCGCTGTGGTAGCAGCCCTCATCGTGGT
GGCGGTCATCTTTGCCATCACCACGTGCGCCGTCCGCTCCAAAACG
TACAAGATGGACGGCCGTCAGCCGCTGCTCGGAGAACAGTACCAGC
GCTACGATGATCACGACAGACACAGCGACAAGACCCAGTCTGTGGTT
TAAAACCACTGGTGCTAGAAAACCCAACCACTGCCCTCTATCTCTCAG

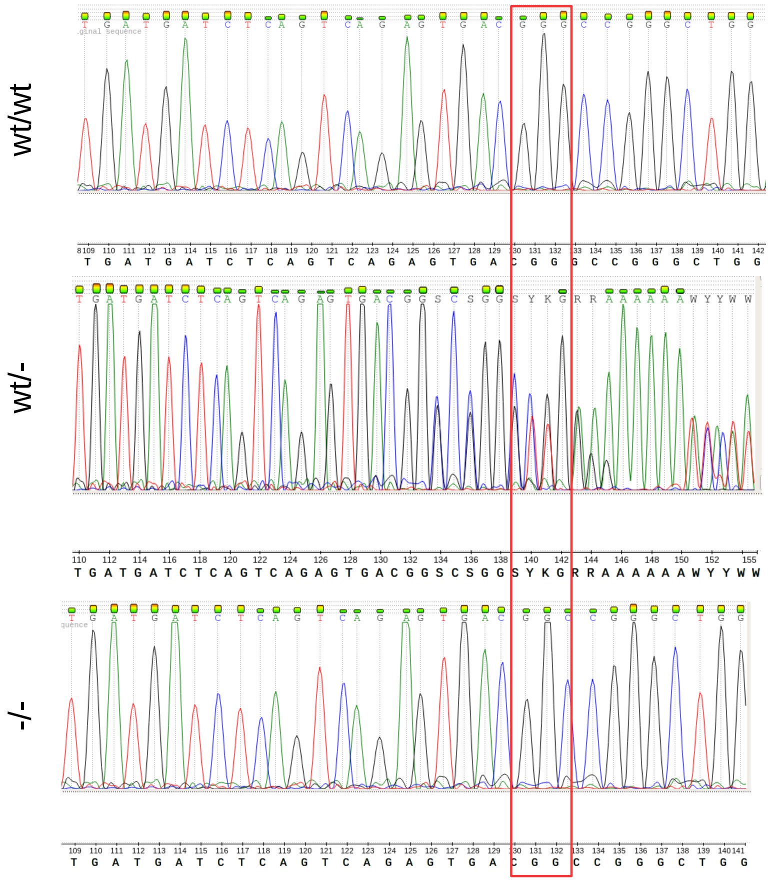

pde6b

11bp deletion

GAGTCCTAATGAGTGGGTTGGAAAATTGGATAAAAATTTAATAGGAATAAAATGAT
TTTTAAAGACTGTGCACTCTTGTTGATGCGTGTTTGTTGGTGTATTTCTCCAGCC
CAT**GATGGACCGAAACAAAGCAGCAG**ACCTTCCCAAGCTTCAGTGCGGTTTC
ATAGACTT**CGTCTGCACTTTCGTCTACAAGGT**ATTTCTCAGCGTTTTTCTATATT
GATGGTTTTAAAACTCCTAAAGCCAGCATAATATGAACCTTATATTAAACTGTTCC
TGTGCAGGAGTTCTCTCGCTTCCACCCGTCCATCCAGCCCATGTATGACGGCA

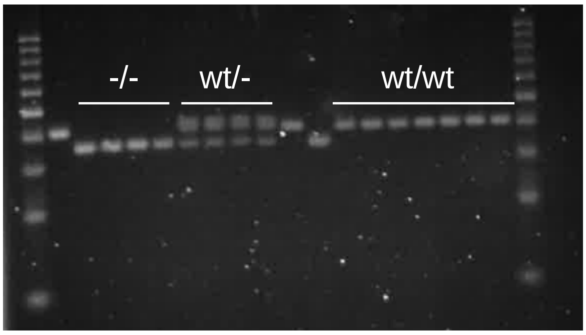

cryba2a

Stop codon (TAT → TAA)

GGCTTACTTAGTGTCTCAAAAAAATTGAATATTTTAAGACCAACTGGTA
CTTTT**GGCAGTGTCCAGAAGTAAACCTTG**ATCCTAGTGTTGATGCTT
GTGAAACAGCAGCGTTGTTCTGATGGCTTGTTCCTCCTGGCTTCTCT
CCAGCTGGGTGGGTTATGAGTATCCAGAGCACCAGGGCCAGCAGTT
CATCCTGGAGAAGGGAGACTATCCCTGTTATCAGGCCTGGAGTGGC
AACAGCAGCTACCGGACCGAA**CACCTGCTCTCCTTCAGACCAAT**TAA
GTGTGCTGTAAGAGCCAACCATACCGTACACCTTTTTCATTAAGGGCT

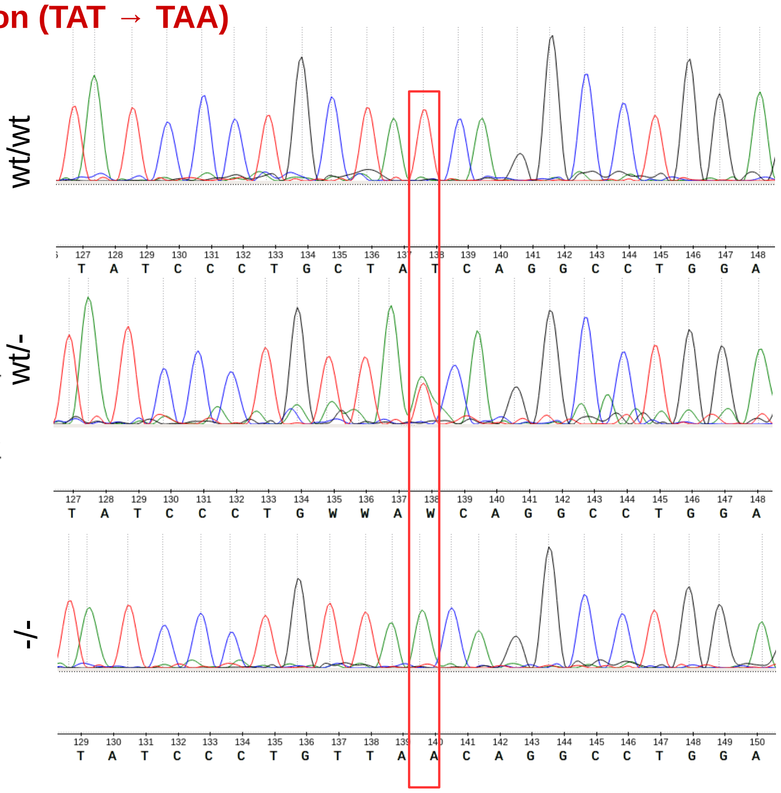

impg1b

13bp insertion

TTTGGACTGTAATGAATTCTCCCGCTGTGTCGTAAACACACTGACTGCAGAAGCTGAGTGTTTGTGTG
ATCCTGGCTACAGCACGGAGAACGGCCAGCCCTGTGAAAGCATCTGTA**ACCTCCAGCCTGACTACT**
GCCTAAATGGGGGGACCTGTGAAATCCTAGCA**NNNNNNNNNNNNN**GGCCATG**GAGCCACTTGCAG**
GTACCTCCATACTGATCAGGTTTTTTTTATATACAGCTTCGGAAAAATTAAGAGACCGATTTCTGAATC
GGTTTCTCTGATTTTGCTATTTATAGGTTTATGTTTGAGTAAAATGAACATTGTTTTATCTTATAAACTAC

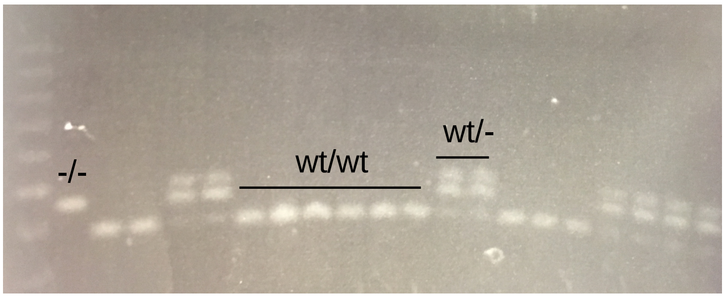

gja8a

16bp deletion

CTTAGGTAATATTTTAGAGGAAGTGAACGAACACTCGACTGTGATTGGCCGCGTTTGGC
TCACGGTGCTCTTCATCTTCCGTATTCTGATTCTGGGCACGGCGGCGGAGTTCGTCTG
GGGCGACGAACAGTCCGATTACGTGTGTAACACC**GAGCAGCCTGGTTGTGAGAA**TGT
TTGTTACGATGAAGCT**TTCCCTATCTCTCACA**TCC**GGCTTTGGGTTCTGCAGATCATC**T
TCGTTTCCACACCTTCGCTGGTCTACGTCGGCCATGCTGTCCATCACATGCACATGGA
GGAAAAGCGCAAAGAGCGGGAGGAAGCAGAGCTGAGCCGGCAGCAGGAAATGAACG

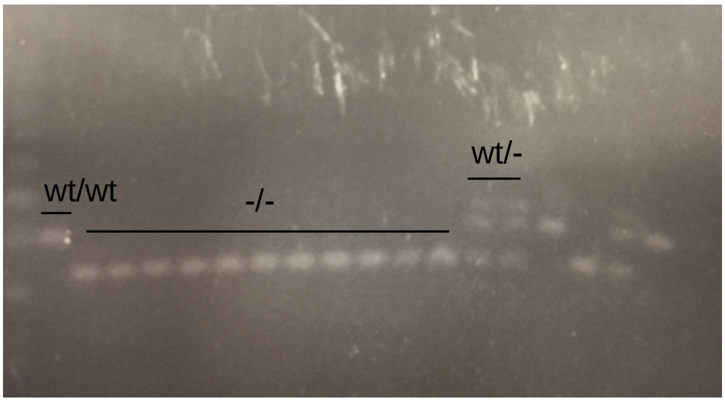

zgc:153441

2bp insertion

AGTGATTGTATTTGTTTTAATGTCACCCTTATAACTATTGTTAATGTCATACATGAACAAGACATTTTTTTATGTCA
AACACTTCCTCCTATCTTTTTTTCTACAGGAAAGATCCATTTTGATGACCTTAACTTCAACAAAACACCATATAAT
TCCTTGATCAGTTACAGGCAGAGCAAACTAGCCAACGTTCTGTTCACCAGAGAGCTCTCACGGCGGATCAAA
GGTTTATATTCTTCTCCCTACAAACTGTACAGAAAAAAAATCAATTTGCCTTTAACATGTTCACCACTCTTTTTTG
GTCTTTTTGGTAGTTTATAATTTATTTGCTCATATGTACCTGTTGATTTTCTGGGCAGGGTCTGGAGTAACAGCC
TTTGCCGTGCACCCTGGAGTGATCCGCACTGAGCTGGGCCGATATGTGGAGATGCGCCATCCTGTGGTCAG
TACCCTGCTGTCCCTTCCTGCCATCCTCCTCATGAAGACCCCGAAGCAGGGAGCTCAGACTACCATTTACTG
CACCATCACTGAGGGACTGGAAAAATACAATGGCGCTGTTACTTTAGGTAAGTTCATAAAAATCCAAAAGTGA
GTATATATTTATACAGTTTCAGAAAAAATTAAGATACCACTTAAAAATGCTAAGCTTTTTTTGATGGCTTGTGACC
ATCCATCTTCCTCTTGACAGAGGTTTAAATAGGGTTGGTGGATGGTCACAAGCCATTAAAAAAAGCAGGACTG
CTTGAATTTTAGCACAAAGAGAGGCAAGTAATGATAAAATTTATAAGTTCAGTCCTAGTTTATAAGATACAGAAA
AAAAAATAAAAAGCATTTTTATGATAATGATCTTTGCATCTTTGGTAATGGCAGTGACTGCCGGATGATGGAGCC
AGCTCTGGAGGCTAAAGATGACCTTGCTGCCATGAGGCTGTGGGATGTGAGCGCTAAGCTGGTGGGCTACA
AAGAAGAG

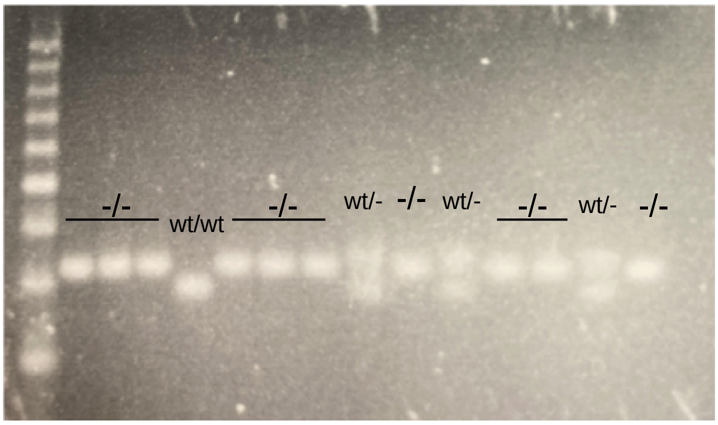

prph2b

Stop codon (AAG → TAG)

GTGGATCAACAACAAAGTAAACAAAGGAAGATTCTGAGGGCATGAAAAAAAAAAAAGAGATCAGA
TTATAGGTCATTTGATTAATTAGGTTAGGTCCAAGTCCAAGGTTTAACAAATAGACTGCAGATATTT
ACCAATCCATCATTTTTTATTTTTGTTTGTTTATTTGATAAACAGATAGCAGATATGGT**TGGACTGA**
AGTACCTGACAACCTCTTTGGAGACCTTGGAGAACCCAGAGAACCCAGAAAGCGAGAGTGAG
GGTTGGCTGCTGGAG**A**AGAGTGTGAAGGAGACCATGTCTGGCATCATGGAGAAGATCAAGACC
TTTGGCAAGGGCAACCAGGTGGAGGGGGAGGGGGCA**GAAGCTCCTGCCACAAGCTGA**GATGA
TCACACCTTTAACTTTGGGACTGTTAAAGCTTAATTGAGAGAGAACAATAAATTAAAGTTATTCTC
TCTGTGGAAAACACATGCACAAATTTCATTAATACAAACCCACAGGTGCACATCCACATACTGTA

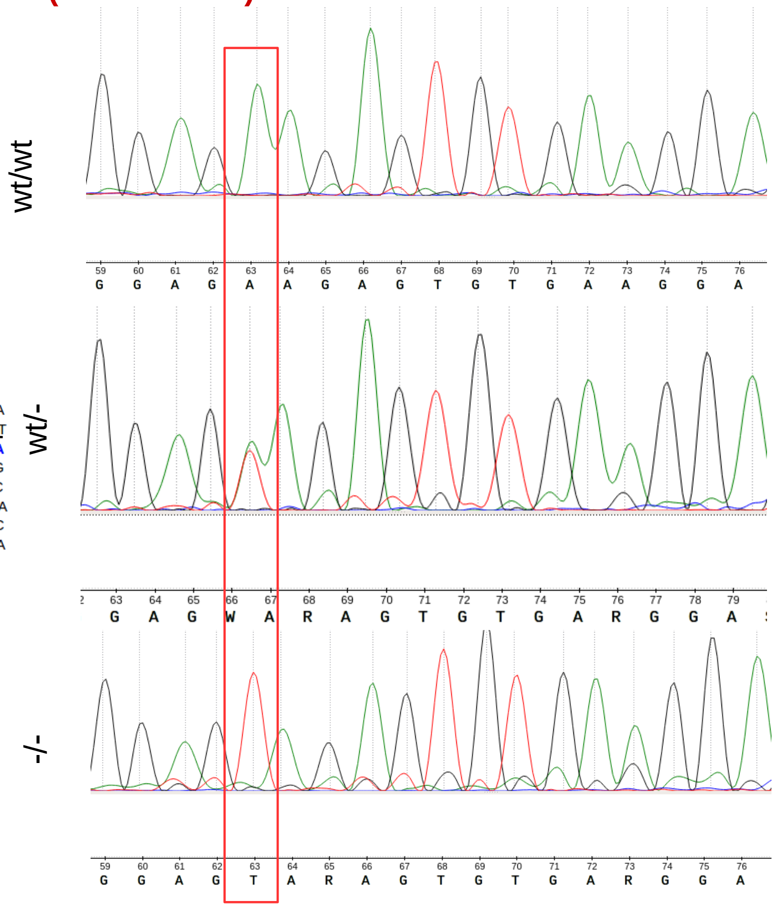

lim2.1

7bp deletion

TTCACTCTGAAGTAAAAATAAATTAATTAATTAATAATAAAAAAAATGGTACTATTATAATACAAAAAA
ATCGTAAATATCGTTCGGACTAAACCGACCAAAATATTAATCTGAGTGTAAGCTGTGGTGTGTGTT
TGTGTGCAGGCTTTTTCGT**GTTCTTGGCCATGGCTGTGTAC**ACGGGGGTAACTATGAATTATTAC
GGTAAGCGCTACGGCAGCTGGCG**CTTCTCCTGGTCCTACATCATCGG**GTGGGTGGCGGTGGTG
CTCACCTTCTTTTCAGGTAGGCTACCTCCTCCGCACACAACCGCCCAAGTGAAGAGTGTACTCT
CTGTCTGATATTTTATTATCCTTTATATTGATTGTTTTAAAGCTGAATGTGCTTCTCCTGCTCTTCCT
CTGTTGCAGGTATTTTCTATATGTGTGCCTACCGGATGCATGAATGCCCCAGAAACCCAGCTCCT
CACTAGGAGGCGAGATGATGAGAT

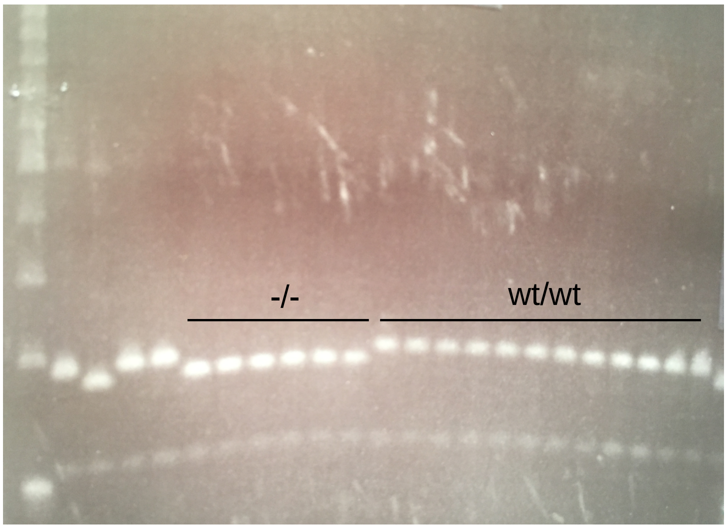

Per2 (E14)

7bp insertion

CACCTGATGAGCGTGGCGTCATCCAGCGAGAGTAACGGTAACGGAACTCGCCCGCACTTCGA
GGACGAGGAGAACAGCAGAGCCAAACCTGTGAGTCACATATTACTATTA**CCTGACTTCATCAT**
CTGTCACATGACTTTTGGTCAGCATCTTTCATTAGAATTTTCTCATCACCATTTTCTTTCACATTA
GGGCACAAAAATCGGACTTGTCAATATAACATTTCAATATAAAGATTTTAATGAAAAAAACATTGT
AAATAAAATTGAAAAAATGGAAGCACCCATTTAAAGATTAAATAACTTTATATTAATGTACAGATT
CCATACCAGTCTCATGTTTCAGGGCCCTACATTCCTTAAATAGTTATCTACTACTCCAGCACTGT
TTGTATAGAGCATGATATGAATAAGTGTCTGTGTGTTTCTACAGAGAACCTTTCACCTTTCAGGA
GATCTGTAAGGGTGTTCACATGCAGAAGAACCAGGAGCAGCAGACCAGAAAG**GCTCCAGGC**
AGTAAGTCCATAGATAAGATTCCATATTCCATGTGCTTAGATATCTGTCTGTGTATAAAGGTGTC
TGAGTTAGGTATAAATGTGGGATACAGAAATCTAAAGTGTTTTTCACTCCTAATTAGTTTGGTCT
GACCAAAAGAGATTACTTACCCACTTAGGCTGGATCAGCTGAAGCATGGTCTGCTTCATCTGC

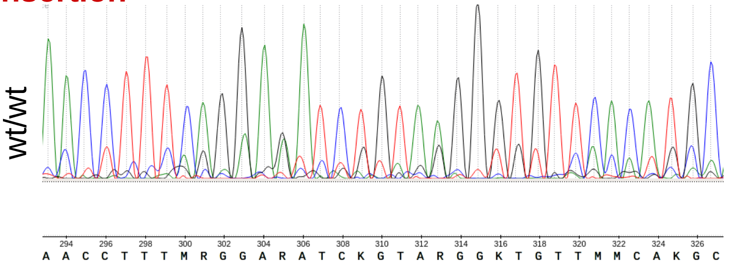

No heterozygous detected

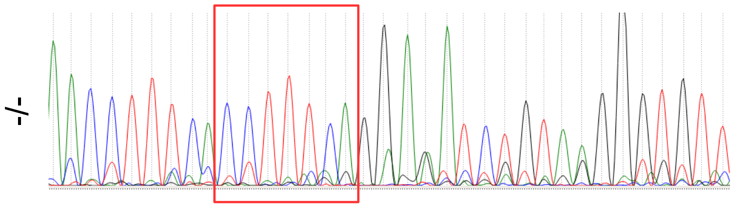

Per2 (E19)

2bp deletion

CTACCCTGAACAAGGAACCTTTGCTGCTCAGCCATCATTTCCCCCACAGACTGTTTTCCCTCCC
CAGACCTCGTTTCAGCCACAGACGACCGTACAATTT**CCTGTACAGACTCAGTTCACCAC**GCAA
AACCCTTTCCCCCCGCCCACGACCTTCCCCACCCAGCCCTTTCAGTTTCCTCTCCCAAACGAT
CCTCCAAAGCCCATGGAACCAGAGCTGAGGGAGGCGCAGTCACGGAGCTCCACCCCTCAGTC
TATGGGTGGGCGGGACCAACCGTCTCCGCCTCTTTTTCAGT**CA**CGGTGCAGTTCGCCCCTGCA
GCTCAACCTGCTGCAGCTGGAGGAGAGCCAACGGTCTATGGAGAGACAGGACAGCTCGGCGC
CCTCTACTGGAGCTCAGGGGAACACCAGCACTGCAGCAGAGAAGACTCTGGC**CAAGACTGAC**
AAGGAGCAGGTATGGAATTTCATAGGGTGCCCTGTATTAAGCGTTGAGTCTCTGGCTGAATCCC
AAATGGCTCCCTACTTGGTTTTTGGCTAATAGGTTACTTTGTTTTGCTAATGCTGTGATAAATCATA
CTGAAGCCTGCCCACCCTAAACATCCTTAACATCCTAATTCTAAAAGTAGAAGTTTAAAAGTGTT

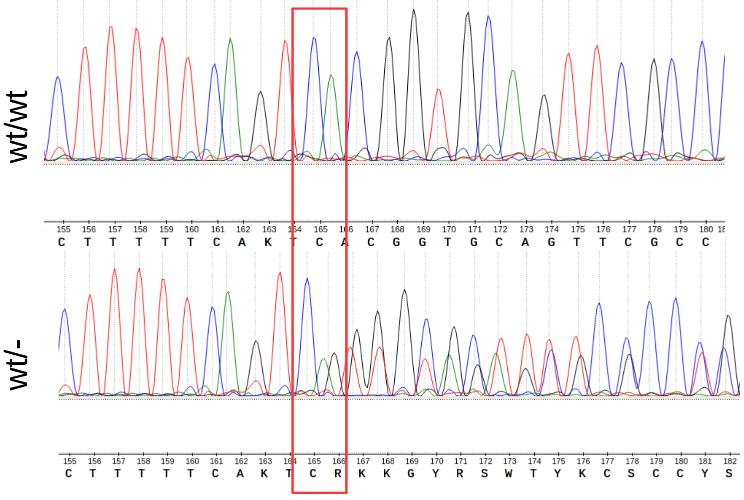

No deleted homozygous detected

Expected gel pattern

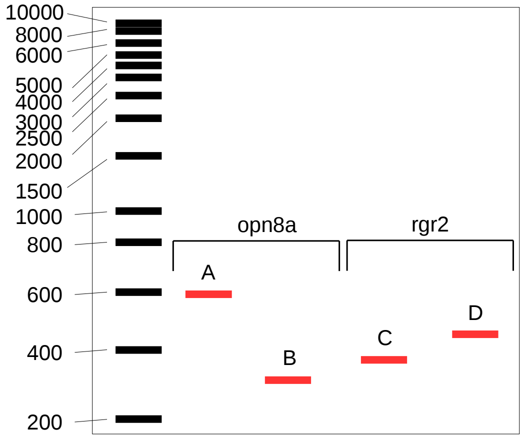

A – opn8a – exon 1 (positive control)
B – opn8a – exon 4 (deleted in some cavefishes)
C – rgr2 – exon 3 (positive control)
D – rgr2 – exon 5 (deleted in some cavefishes)

opn8

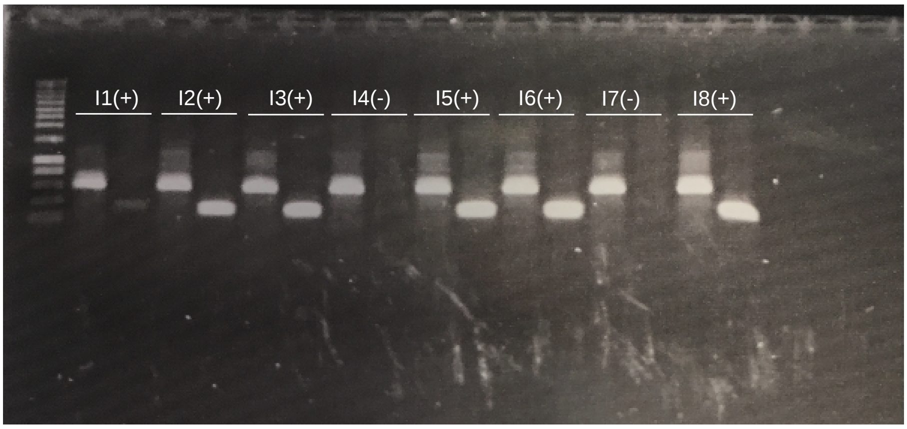

I1 – PCF3-A4 (Chica)
I2 – PCF3-B4 (Chica)
I3 – PCF3-C4 (Chica)
I4 – PCF3-D4 (Chica)
I5 – PCF3-E4 (Chica)
I6 – PCF3-F4 (Chica)
I7 – PCF3-G4 (Chica)
I8 – PCF3-G4 (Chica)

rgr2

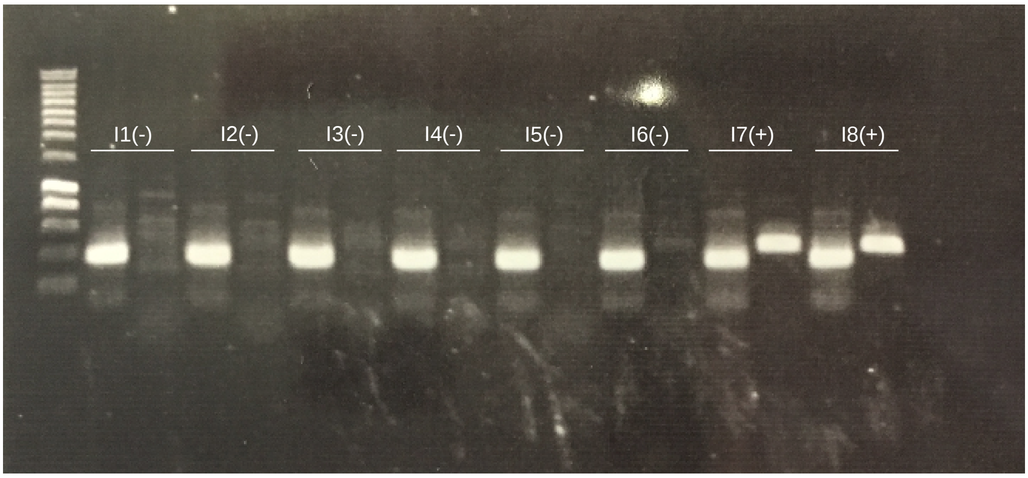

I1 – PCF1-A8 (Escondido)
I2 – PCF1-B8 (Escondido)
I3 – PCF1-C8 (Escondido)
I4 – PCF1-D8 (Escondido)
I5 – PCF1-E8 (Escondido)
I6 – PCF1-F8 (Escondido)
I7 – PCF1-G8 (Sabinos)
I8 – PCF1-H8 (Sabinos)

Additional file 2_Fig S7:

(A) Representation of deleted regions in the cave populations. Exons of genes are represented by blue squares, and exons amplified to genotype these deletions are indicated either with blue lines (exons used as control) or red lines (exons in the deleted regions). The deletion region in cave individuals is represented with dotted lines.

(B) The left panel show the expected gel pattern if an individual is not deleted. The right panel show a gel with homozygous deleted individuals (-) and with non-deleted individuals (+) for which we cannot differentiate between heterozygous or homozygous non-deleted.

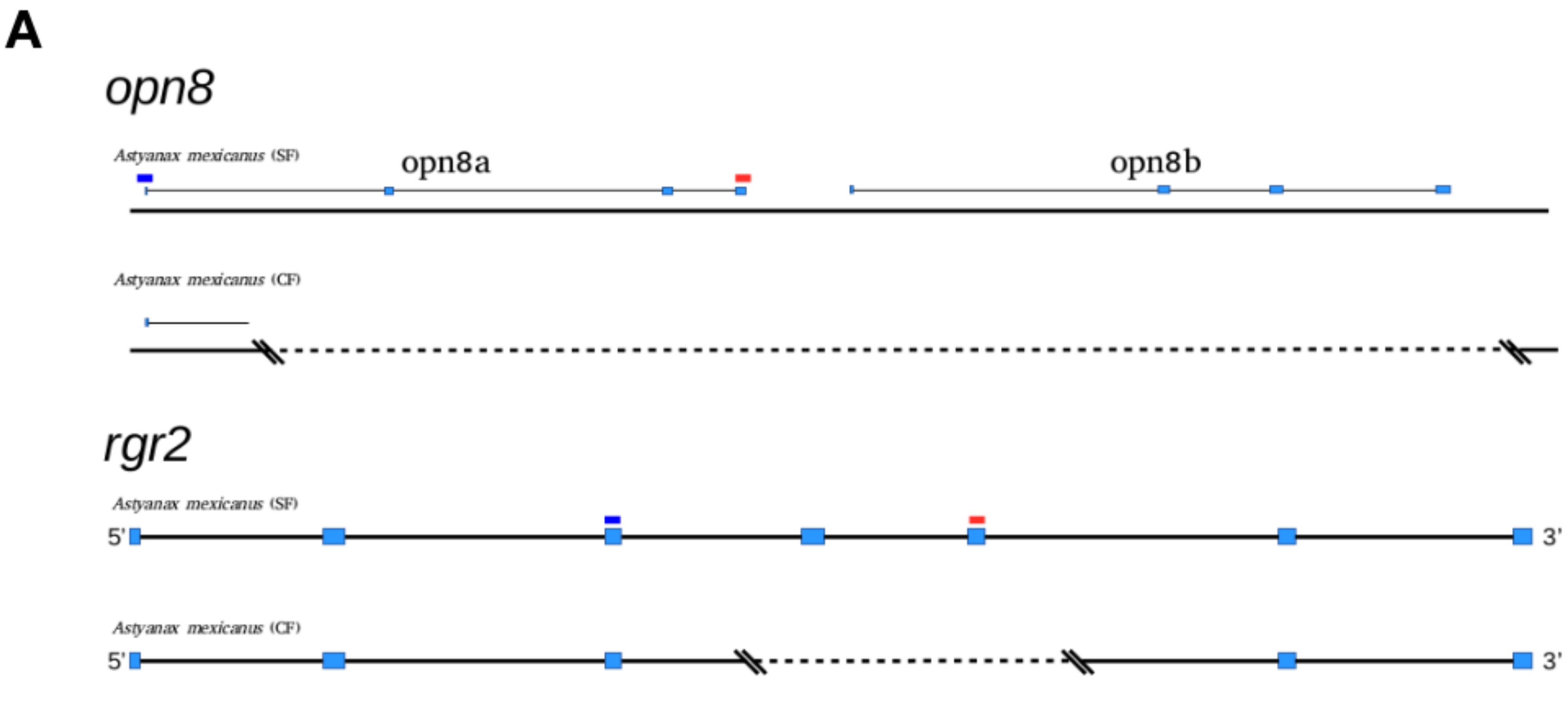
A
opn8
Astyanax mexicanus (SF)
opn8a
opn8b
Astyanax mexicanus (CF)
rgr2
Astyanax mexicanus (SF)
5'
3'
Astyanax mexicanus (CF)
5'
3'

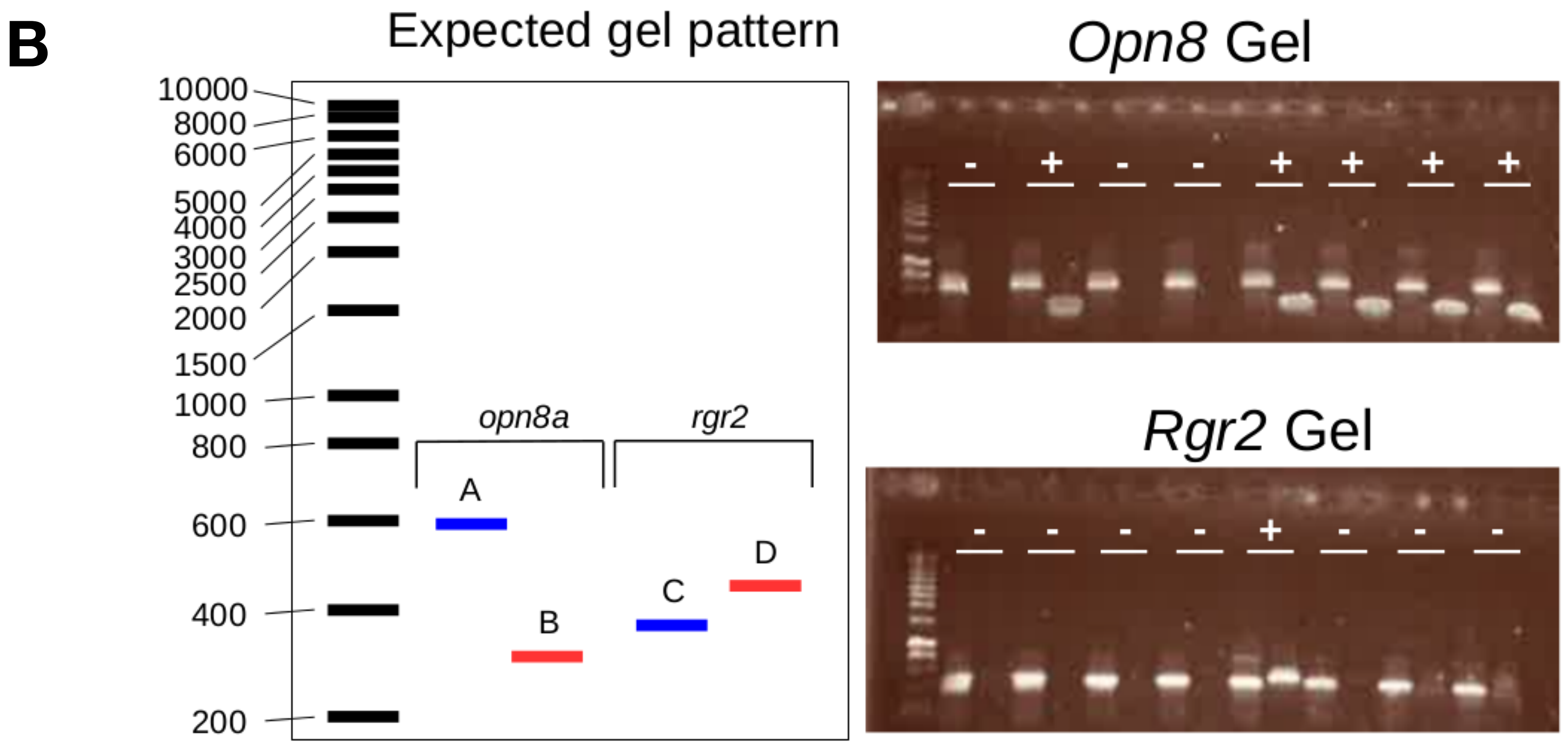
B
Expected gel pattern
10000
8000
6000
5000
4000
3000
2500
2000
1500
1000
800
600
400
200
opn8a
rgr2
A
B
C
D
Opn8 Gel
- + - - + + + +
Rgr2 Gel
- - - - + - - -

Additional file 2_Table S1:

(A) GO-term enrichment analysis results with all human orthologous genes. Only significant GO-terms are shown.

(B) GO-term enrichment analysis results with all zebrafish orthologous genes, using the "GO Biological Process GeneRIF Predicted Z-score" database. The 25 top results are shown, but neither of those are significant.

(C) GO-term enrichment analysis results with all zebrafish orthologous genes, using the "Phenotype GeneRIF Predicted Z-score" database. The 25 top results are shown, but only the 21 first terms are significant.

A - 335 *Danio rerio* orthologous genes - GO Biological Process GeneRIF Predicted Z-score

Index	Name	P-value	Adjusted p-value	Z-score	Combined score
1	eye_photoreceptor_cell_differentiation_(GO:0001754)	0.00003454	0.01899	-2.65	27.22
2	peripheral_nervous_system_neuron_development_(GO:0048935)	0.00003454	0.01899	-1.79	18.41
3	cellular_response_to_UV_(GO:0034644)	0.0001474	0.02026	-3.53	31.13
4	response_to_UV_(GO:0009411)	0.0001474	0.02026	-2.95	26.07
5	eye_photoreceptor_cell_development_(GO:0042462)	0.0001474	0.02026	-2.68	23.66
6	retinal_rod_cell_development_(GO:0046548)	0.0001474	0.02026	-2.65	23.40
7	retinal_cone_cell_differentiation_(GO:0042670)	0.0001474	0.02026	-2.65	23.40
8	photoreceptor_cell_development_(GO:0042461)	0.0001474	0.02026	-1.80	15.88
9	retinal_cone_cell_development_(GO:0046549)	0.0005780	0.04542	-2.69	20.07
10	phosphatidylinositol_metabolic_process_(GO:0046488)	0.0005780	0.04542	-2.07	15.45
11	phosphatidylinositol_biosynthetic_process_(GO:0006661)	0.0005780	0.04542	-2.06	15.33
12	opsin_transport_(GO:0036372)	0.0005780	0.04542	-2.03	15.17
13	photoreceptor_cell_differentiation_(GO:0046530)	0.0005780	0.04542	-1.76	13.13
14	protein_prenylation_(GO:0018342)	0.0005780	0.04542	-1.75	13.05
15	lymphocyte_differentiation_(GO:0030098)	0.002072	0.1266	-2.98	18.40
16	regulation_of_photoreceptor_cell_differentiation_(GO:0046532)	0.002072	0.1266	-2.41	14.89
17	retinal_metabolic_process_(GO:0042574)	0.002072	0.1266	-2.39	14.75
18	pineal_gland_development_(GO:0021982)	0.001997	0.1266	-1.82	11.32
19	response_to_glucocorticoid_(GO:0051384)	0.006745	0.3710	-3.15	15.73
20	mitochondrial_fusion_(GO:0008053)	0.006745	0.3710	-2.52	12.62
21	T_cell_differentiation_(GO:0030217)	0.05190	0.9665	-4.84	14.33
22	cGMP_metabolic_process_(GO:0046068)	0.05190	0.9665	-4.30	12.73
23	cGMP_biosynthetic_process_(GO:0006182)	0.05190	0.9665	-4.17	12.35
24	cortisol_metabolic_process_(GO:0034650)	0.01979	0.9665	-2.59	10.17
25	melanosome_transport_(GO:0032402)	0.05190	0.9665	-3.15	9.32

B - 335 *Danio rerio* orthologous genes - Phenotype GeneRIF Predicted Z-score

Index	Name	P-value	Adjusted p-value	Z-score	Combined score
1	eye_photoreceptor_cell_differentiation	0.00003454	0.008444	-474.39	4873.65
2	retinal_outer_plexiform_layer	0.00003454	0.008444	-329.22	3382.27
3	rhodopsin_metabolic_process	0.00003454	0.008444	-320.03	3287.78
4	photoreceptor_cell	0.00003454	0.008444	-210.00	2157.44
5	photoreceptor_cell_morphogenesis	0.00003454	0.008444	-133.26	1369.05
6	optomotor_response	0.00003278	0.008444	-14.67	151.47
7	basal_protein_localization	0.0001474	0.01272	-494.30	4361.01
8	eye_photoreceptor_cell	0.0001474	0.01272	-472.82	4171.46
9	eye_photoreceptor_cell_development	0.0001474	0.01272	-472.19	4165.91
10	cellular_response_to_UV	0.0001474	0.01272	-442.20	3901.37
11	retina_layer_formation	0.0001474	0.01272	-331.21	2922.09
12	retinal_cone_cell_differentiation	0.0001474	0.01272	-328.83	2901.08
13	retinal_photoreceptor_layer	0.0001474	0.01272	-324.86	2866.07
14	photoreceptor_cell_development	0.0001474	0.01272	-201.00	1773.33
15	retinal_rod_cell	0.0001474	0.01272	-133.01	1173.45
16	retinal_rod_cell_development	0.0001474	0.01272	-84.01	741.17
17	pineal_complex	0.0001474	0.01272	-49.23	434.37
18	regulation_of_circadian_rhythm	0.0005780	0.03141	-558.18	4161.71
19	circadian_regulation_of_gene_expression	0.0005780	0.03141	-449.86	3354.08
20	rod_bipolar_cell	0.0005780	0.03141	-406.88	3033.68
21	retinal_cone_cell_development	0.0005780	0.03141	-343.55	2561.50
22	protein_prenylation	0.0005780	0.03141	-226.91	1691.78
23	protein_farnesylation	0.0005780	0.03141	-216.21	1612.06
24	photoreceptor_cell_differentiation	0.0005780	0.03141	-190.00	1416.62
25	protein_geranylgeranylation	0.0005780	0.03141	-150.69	1123.50

C - 243 *Homo sapiens* orthologous genes

GO biological process / Disease	Database	Raw p-value	Adj. p-value	Genes
visual perception (GO:0007601)	DAVID	1.5E-04	1.9E-01	*ABCA4, CRYBA2, CYP4V2, IMPG1, LRIT3, NRL, PRPH2, PDE6B, RLBP1, RHO, WFS1*
retinitis pigmentosa	ClinVar 2019	3.5E-5	9.2E-4	*PRPH2, ABCA4, SAG, RHO, NRL, PDE6B, RLBP1*
retinitis pigmentosa	OMIM	3.8E-4	8.7E-3	*ABCA4, SAG, RHO, NRL, PDE6B,*
