# Supplementary figures and images for: The nature and distribution of putative non-functional alleles suggest only two independent events at the origins of Astyanax mexicanus cavefish populations
Source: BMC Ecol Evol. 2024 Apr 1;24:41. doi: 10.1186/s12862-024-02226-1 (PMC10983663; doi:10.1186/s12862-024-02226-1)

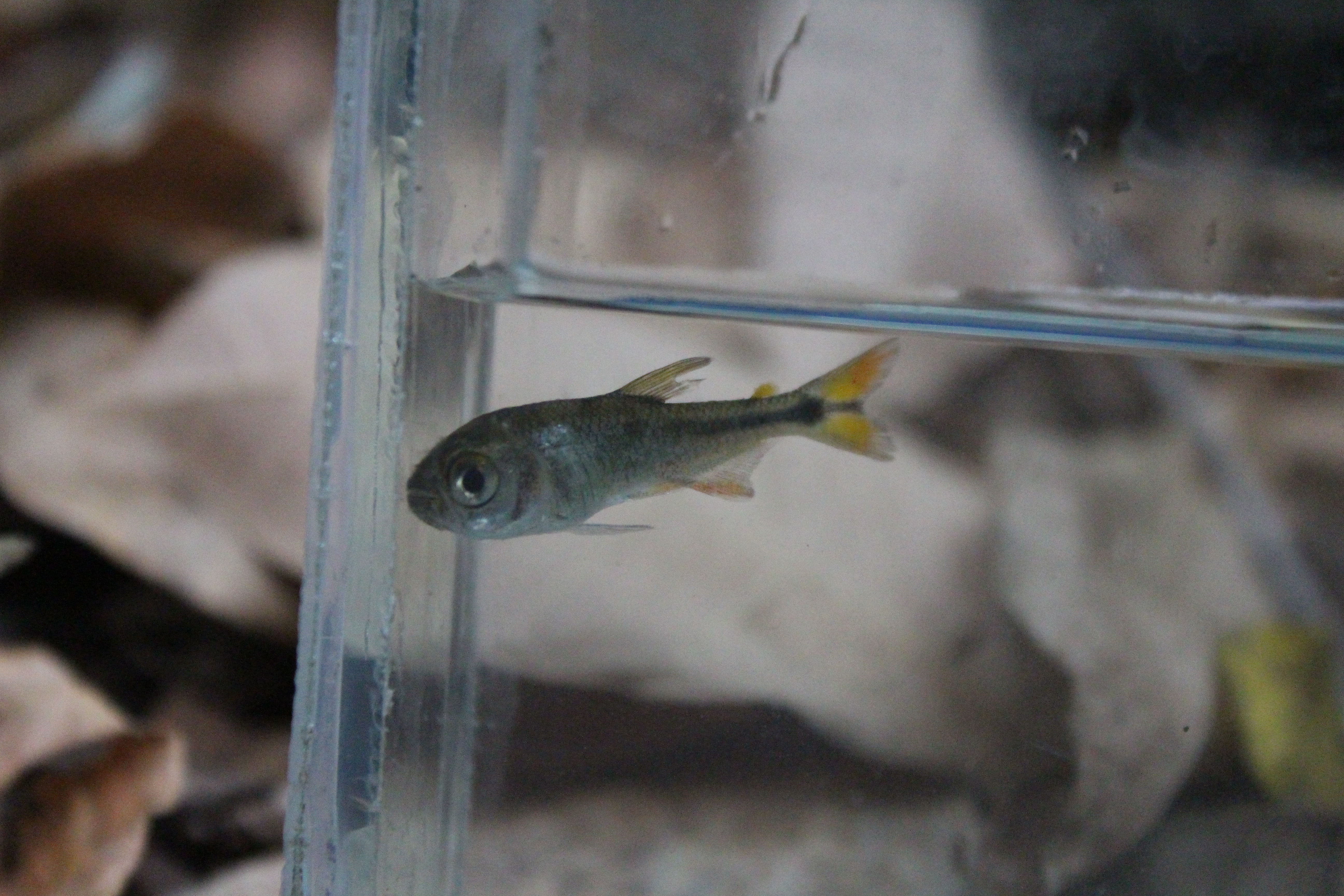

Supplement: Supplementary file 5 — Additional file 5. Original pictures used in Fig. 3. [file 12862_2024_2226_MOESM5_ESM.zip › data_figure 3/Toro A.JPG]

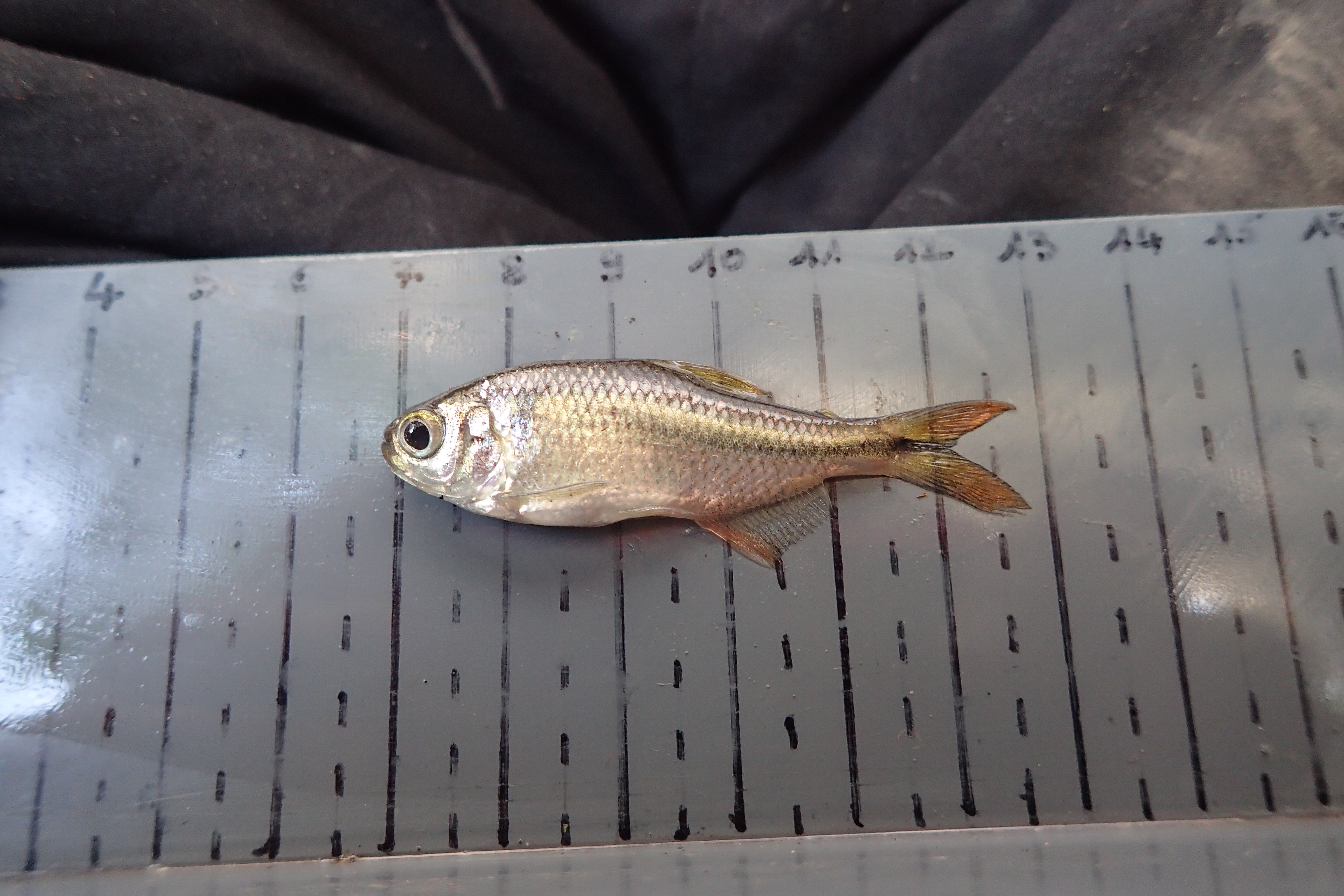

Supplement: Supplementary file 5 — Additional file 5. Original pictures used in Fig. 3. [file 12862_2024_2226_MOESM5_ESM.zip › data_figure 3/Toro B.JPG]

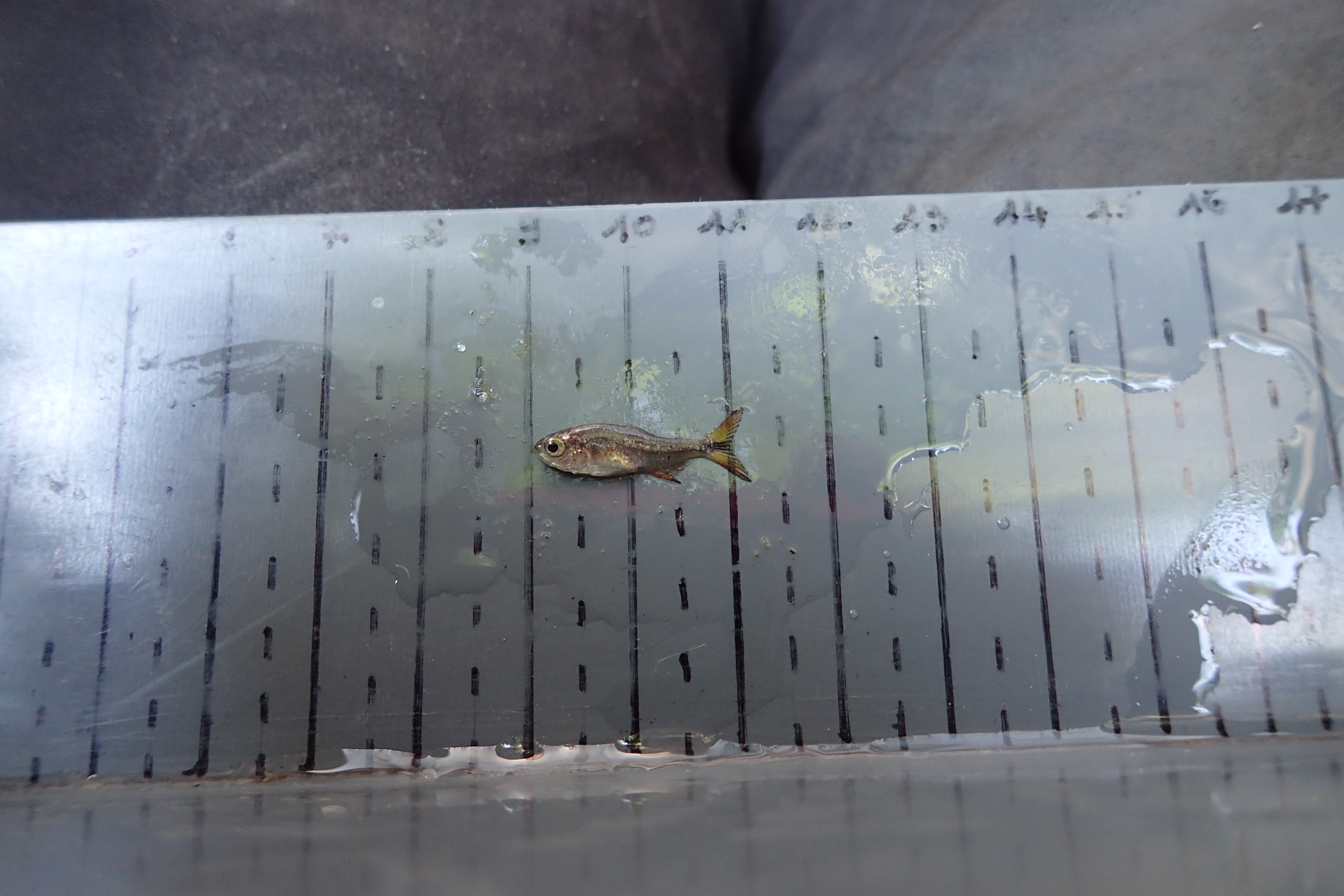

Supplement: Supplementary file 5 — Additional file 5. Original pictures used in Fig. 3. [file 12862_2024_2226_MOESM5_ESM.zip › data_figure 3/Toro C.JPG]

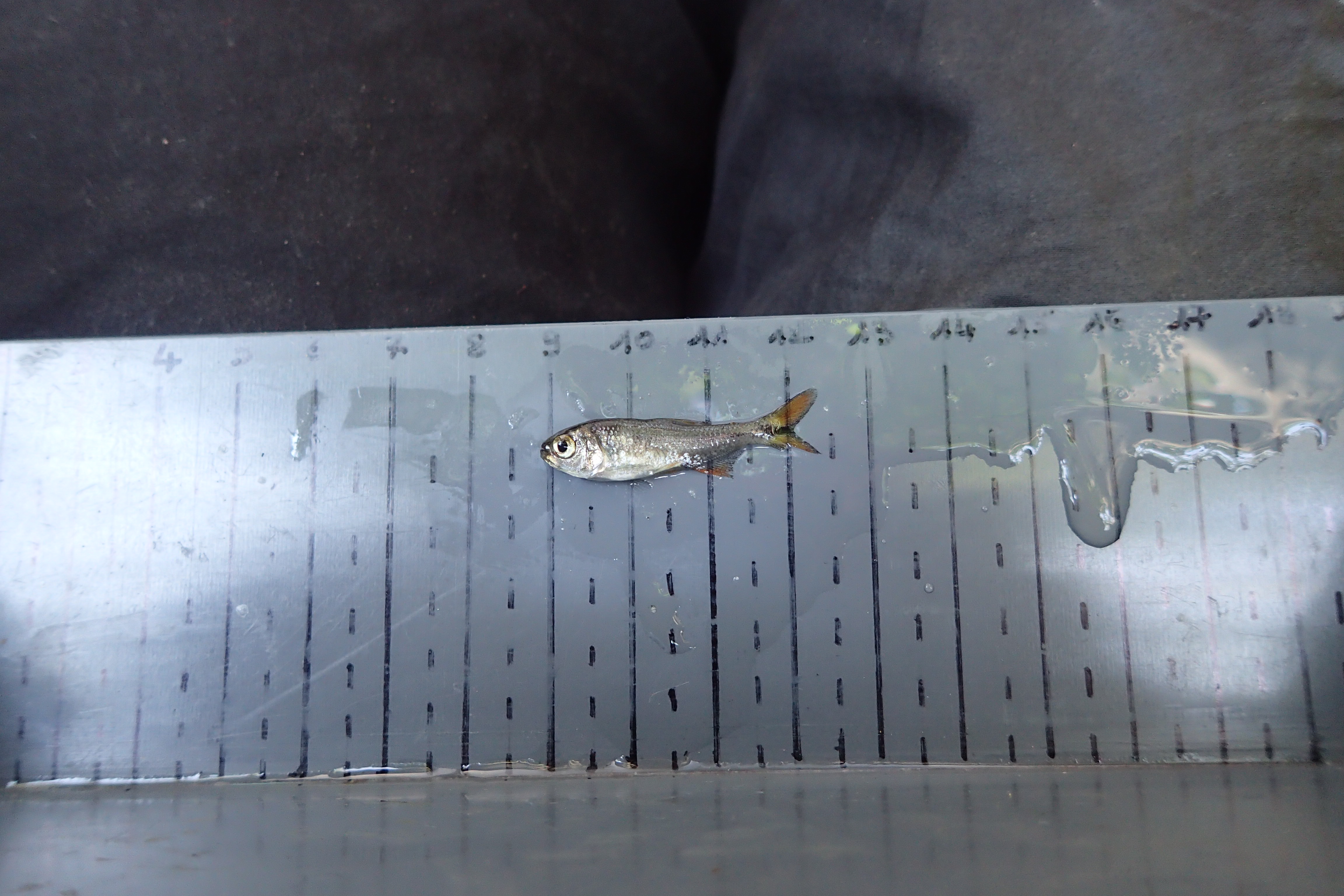

Supplement: Supplementary file 5 — Additional file 5. Original pictures used in Fig. 3. [file 12862_2024_2226_MOESM5_ESM.zip › data_figure 3/Toro D.JPG]

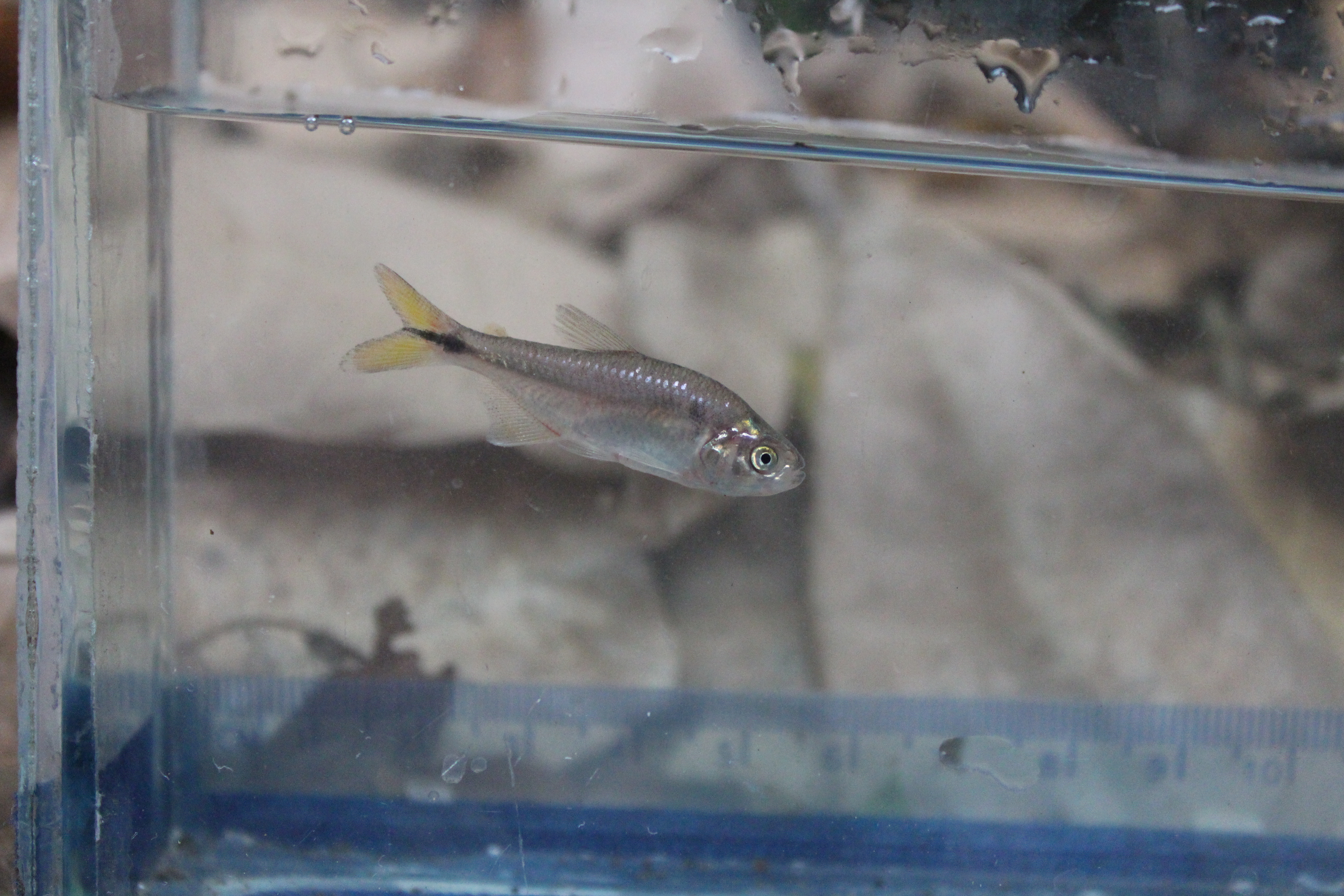

Supplement: Supplementary file 5 — Additional file 5. Original pictures used in Fig. 3. [file 12862_2024_2226_MOESM5_ESM.zip › data_figure 3/Toro E.JPG]

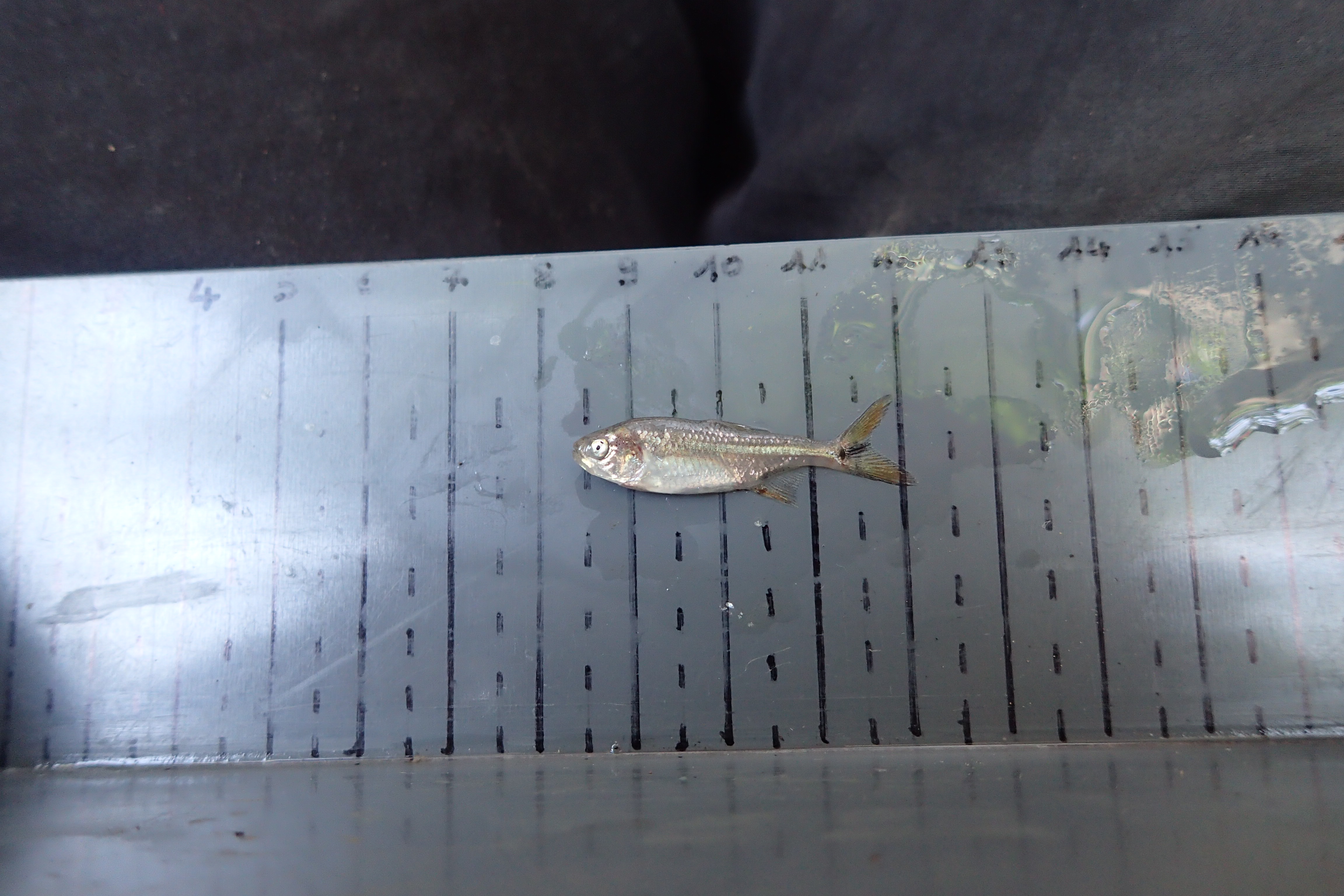

Supplement: Supplementary file 5 — Additional file 5. Original pictures used in Fig. 3. [file 12862_2024_2226_MOESM5_ESM.zip › data_figure 3/Toro F.JPG]

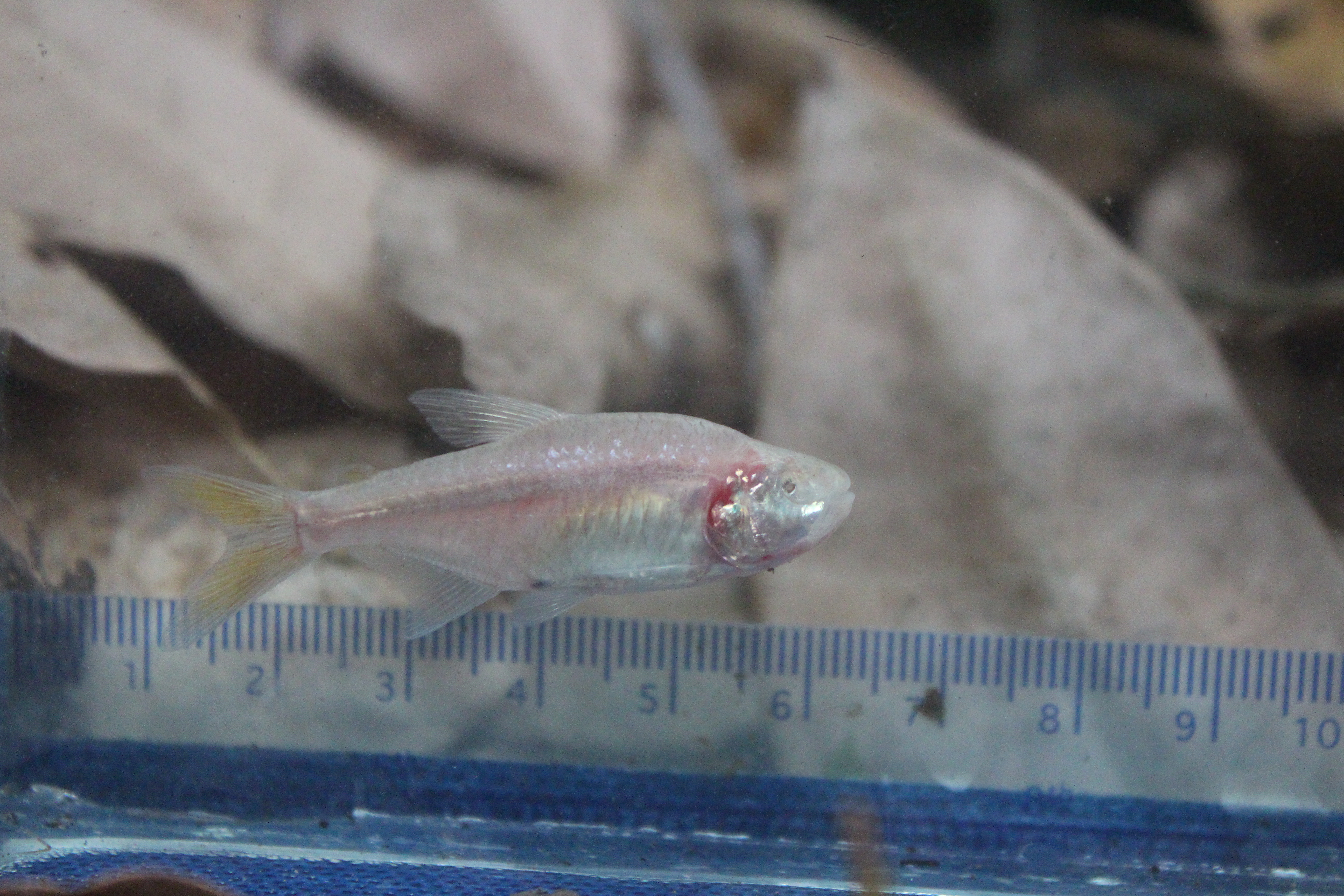

Supplement: Supplementary file 5 — Additional file 5. Original pictures used in Fig. 3. [file 12862_2024_2226_MOESM5_ESM.zip › data_figure 3/Toro G.JPG]

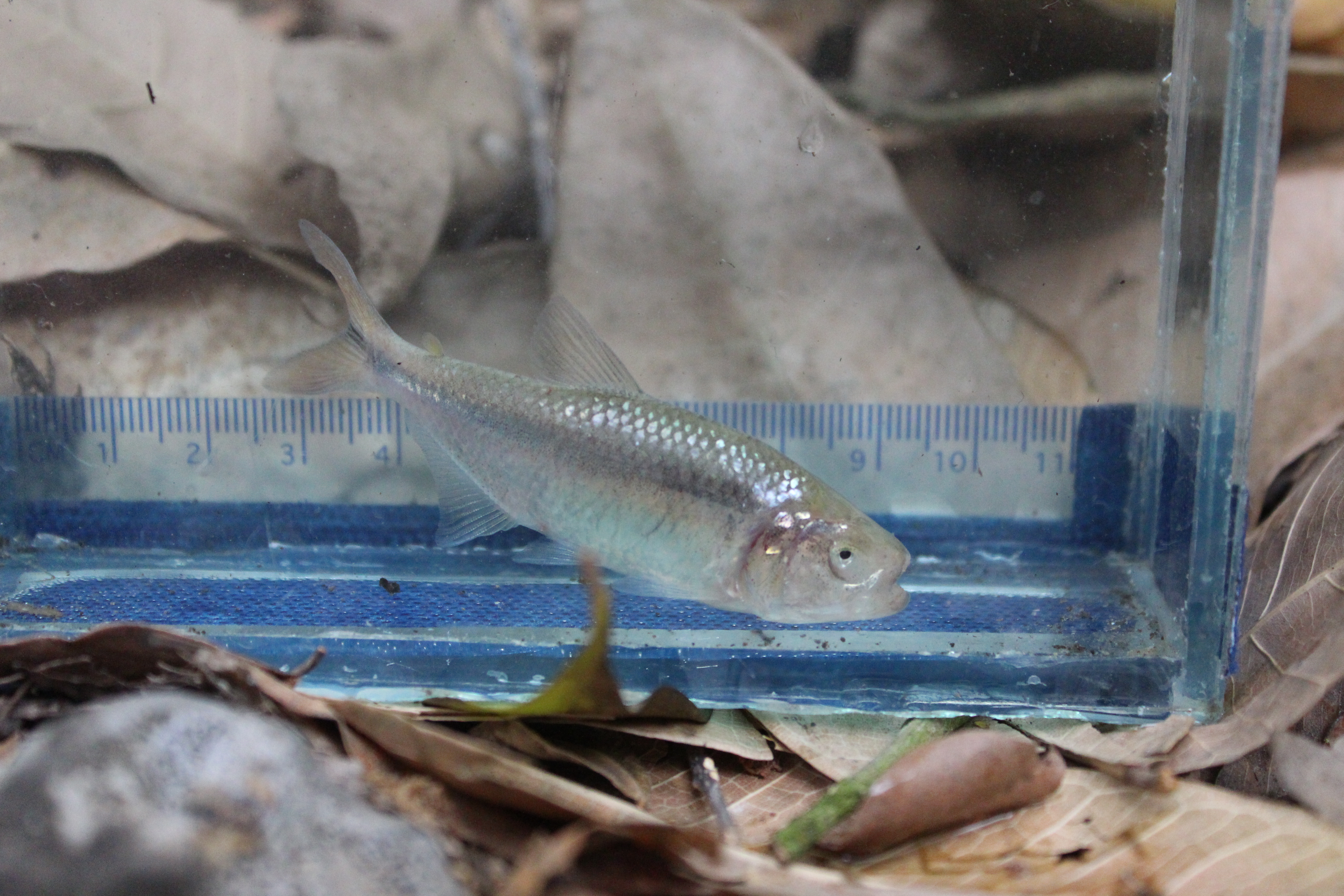

Supplement: Supplementary file 5 — Additional file 5. Original pictures used in Fig. 3. [file 12862_2024_2226_MOESM5_ESM.zip › data_figure 3/Toro H.JPG]

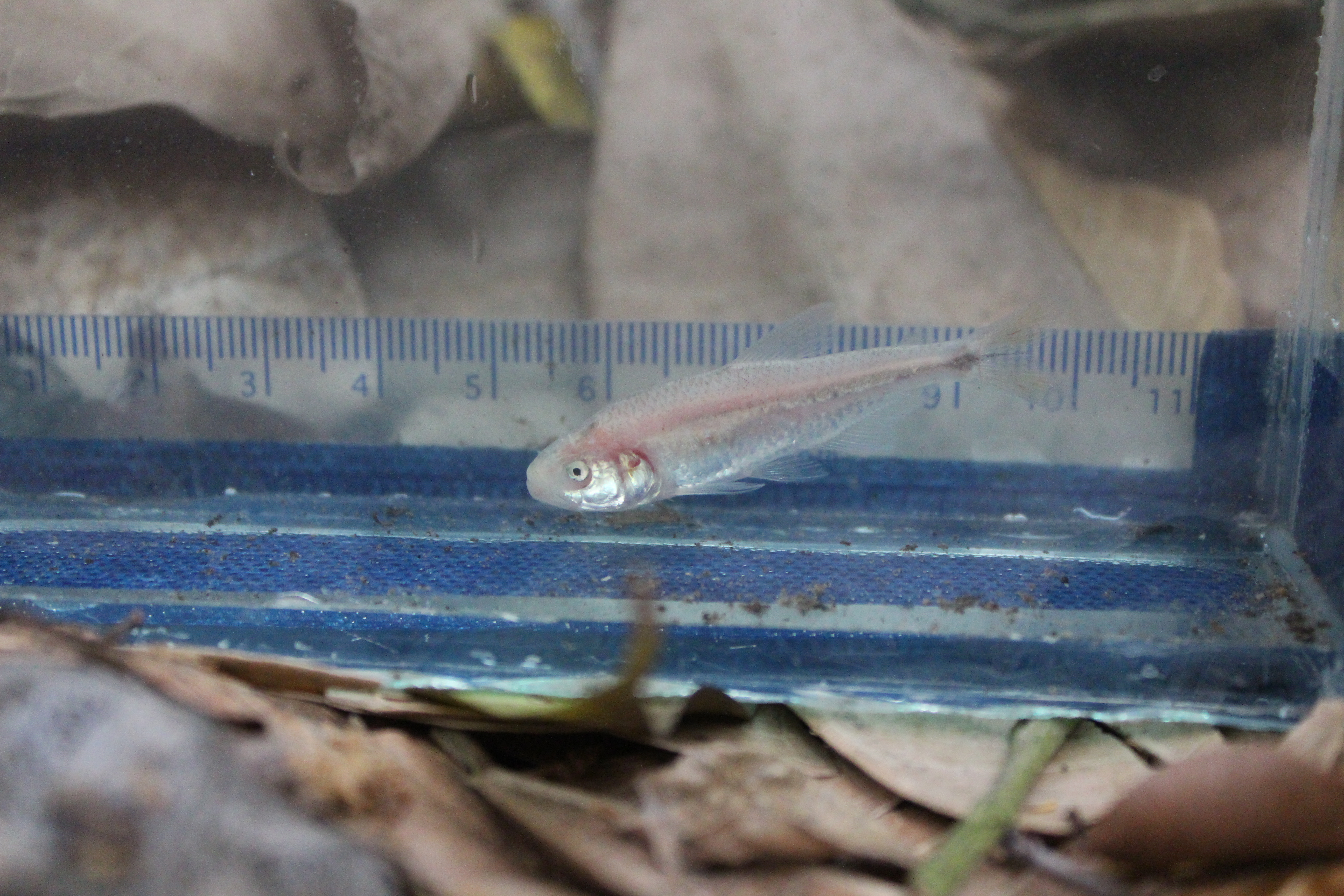

Supplement: Supplementary file 5 — Additional file 5. Original pictures used in Fig. 3. [file 12862_2024_2226_MOESM5_ESM.zip › data_figure 3/Toro I.JPG]

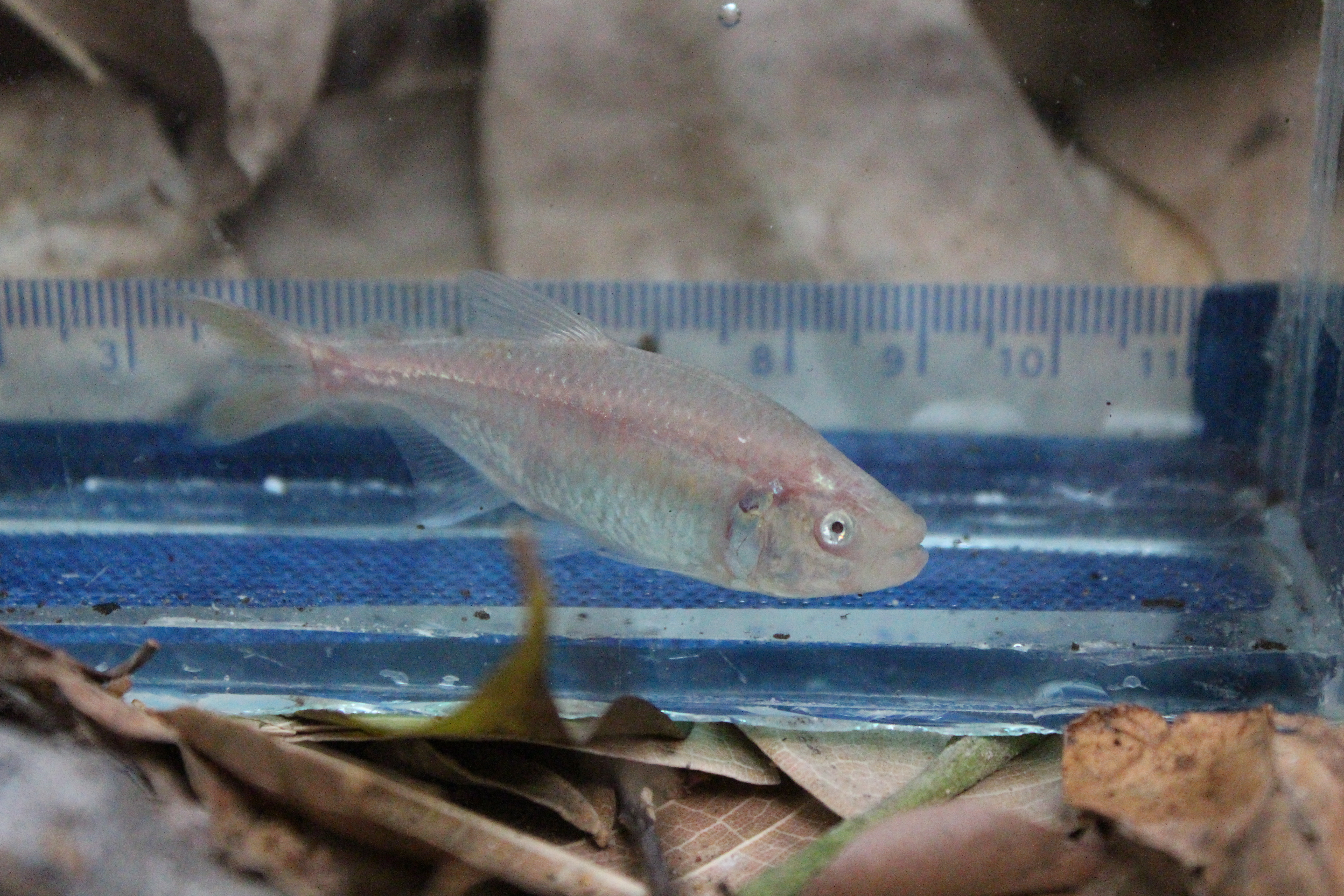

Supplement: Supplementary file 5 — Additional file 5. Original pictures used in Fig. 3. [file 12862_2024_2226_MOESM5_ESM.zip › data_figure 3/Toro J.JPG]

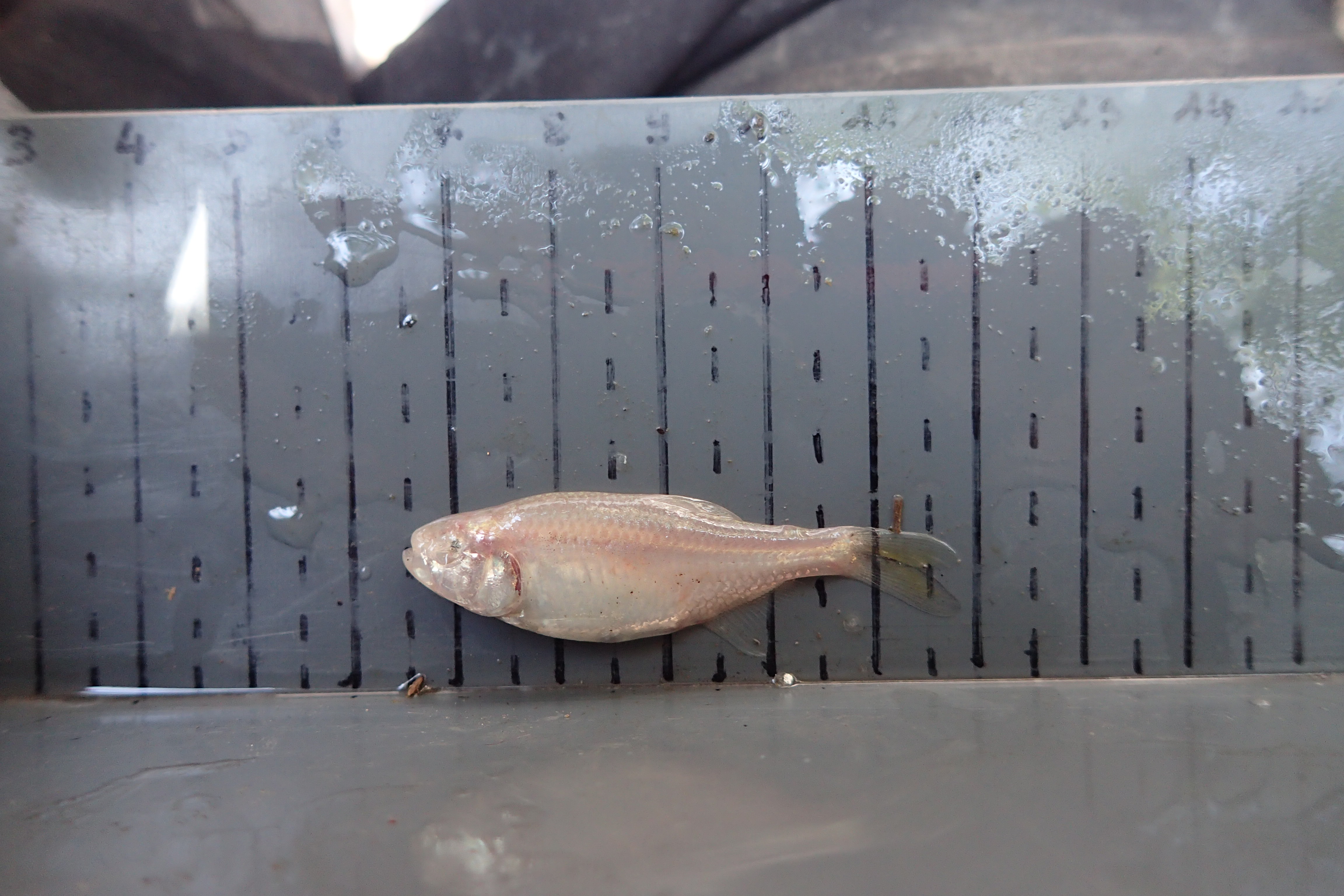

Supplement: Supplementary file 5 — Additional file 5. Original pictures used in Fig. 3. [file 12862_2024_2226_MOESM5_ESM.zip › data_figure 3/Toro K.JPG]
